# Comparison of the efficacy and safety of different doses of atropine for myopic control in children: a meta-analysis

**DOI:** 10.3389/fphar.2023.1227787

**Published:** 2023-09-11

**Authors:** Peixian Hou, Dawen Wu, Yan Nie, Hong Wei, Longqian Liu, Guoyuan Yang

**Affiliations:** ^1^ Department of Ophthalmology, West China Hospital, Sichuan University, Chengdu, Sichuan, China; ^2^ Laboratory of Optometry and Vision Sciences, West China Hospital, Sichuan University, Chengdu, Sichuan, China

**Keywords:** atropine, myopia, dose, efficacy, safety, meta-analysis

## Abstract

**Purpose:** To comprehensively reassess the efficacy and safety of different concentrations of atropine for retarding myopia progression and seek the most appropriate therapeutic concentration for clinical practice.

**Methods:** We searched PubMed, Cochrane Library, Embase, Chinese Science and Technology Periodicals (VIP) and China National Knowledege Infrastructure (CNKI) from their inception to 23 March 2023, to obtain eligible randomized controlled trials (RCTs) and cohort studies that had atropine in at least one treatment arm and placebo/no intervention in another arm. We evaluated the risk of bias of the RCTs according to the recommendations of the Cochrane Collaboration for RCTs and quality of cohort studies by the Newcastle‒Ottawa Scale. Weighted mean difference (WMD), 95% confidence interval were calculated for meta-analysis. All data analyses were performed using Review Manager 5.3, STATA 12.0 and SPSS 26.0 software.

**Results:** A total of 44 studies were included in the meta-analysis. Weighted mean difference (WMD) were 0.73 diopters (D), 0.65 D, 0.35 D per year in refraction progression (*χ* ^2^ = 14.63, *I* ^2^ = 86.3%; *p* < 0.001) and −0.26 mm, −0.37 mm, −0.11 mm per year in axial length progression (*χ* ^2^ = 5.80, *I* ^2^ = 65.5%; *p* = 0.06) for high (0.5%–1%), moderate (0.1%–0.25%), and low (0.005%–0.05%) dose atropine groups, respectively. Logarithmic dose‒response correlations were found between atropine and their effect on change of refraction, axial length, accommodation and photopic pupil diameter. Through these curves, we found that atropine with concentrations ≤0.05% atropine resulted in a residual value of accommodation of more than 5 D and an increase in pupil diameter no more than 3 mm. Higher doses of atropine resulted in a higher incidence of adverse effects, of which the incidence of photophobia was dose-dependent (*r* = 0.477, *p* = 0.029).

**Conclusion:** Both the efficacy and risk of adverse events for atropine treatment of myopia were mostly dose dependent. Comprehensively considered the myopia control effect and safety of each dose, 0.05% may be the best concentration of atropine to control myopia progression at present, at which myopia is better controlled and the side effects are tolerable.

**Systematic Review Registration:** https://www.crd.york.ac.uk/PROSPERO/#recordDetails, CRD42022377705.

## 1 Introduction

Myopia, (near sightedness) is due to the inability of the optical system of the eye to focus incident parallel rays of light on the retinal plane, but instead in front of it. Due to its global “explosive epidemic” among children and adolescents, it has recently become a significant public health concern. High myopia can significantly increase the risk of potential blindness due to pathological changes such as posterior scleral staphyloma, retinal detachment, macular degeneration and choroidal neovascularization (Saw et al., 2019). Early-onset myopia usually progresses more quickly and inevitably results in high myopia. Therefore, it is vital to slow or prevent myopic progression in children.

Atropine has been demonstrated to be effective for myopia control. Previous studies have delved into the optimal dose of atropine for controlling myopia progression. According to these studies, high concentration atropine had a significant myopia control effect, but it led to more adverse reactions such as photophobia, near blurred vision, fever, dry mouth, and even a “rebound effect” after drug withdrawal (Yen et al., 1989; Shih et al., 1999; Shih et al., 2001; Chua et al., 2006; Chia et al., 2014). On the other hand, low concentrations had relatively mild adverse reactions, lower rebound effects after drug cessation and higher acceptance by patients, and can effectively delay the progression of myopia (Chia et al., 2012; Chia et al., 2014; Chia et al., 2016; Yam et al., 2019; Yam et al., 2020). Therefore, 0.01% has long been considered to be the optimal dose of atropine for controlling myopia. However, the recent LAMP study and Ha et al. (2022) network meta-analysis reached the conclusion that 0.05% was the most beneficial concentration with fewer side effects, which refutes the past belief that 0.01% atropine can better control myopia progression (Gong et al., 2017; Yam et al., 2019; Yam et al., 2020). Hence, the optimal dose of atropine for myopia control is still controversial. Considering newer studies like phase three trial of LAMP (which was not included in the previous meta-analyses) supported 0.05% atropine may be more effective than 0.01% atropine in long-term myopia control with small difference in rebound effect (Yam et al., 2022), and other new trials had evaluated the effect of Atropine 0.005% eye drops (Zhao et al., 2022) and included different races (Lee et al., 2022; Nucci et al., 2023), we need to further comprehensively evaluate the efficacy and adverse effects of different doses of atropine in myopia control. On the other hand, how to balance the increasing myopia control effect with higher doses against side effects is still an important issue. Although the previous meta-analyses have focused on the changes in accommodation and pupil diameter (Chen and Yao, 2021; Gan et al., 2021; Tran et al., 2021; Ha et al., 2022), which are two important parameters that affect the compliance with atropine use, they had not combined the clinically tolerable accommodation and pupil diameter changes to select the optimal dose of atropine for myopia control.

In this meta-analysis, we aimed to reevaluate the overall efficacy and safety of different doses of atropine with more updated studies. We also explored the non-linear dose‒response relationship between different atropine concentrations and their treatment effect on some main outcomes (refraction change, change in AL, change in amplitude of accommodation, and change in photopic pupil diameter) and combined these parameters with the clinical experience based on daily visual tasks to seek the most appropriate therapeutic concentration for future clinical research.

## 2 Methods

We registered this study at PROSPERO prior to the start of the data analysis (CRD42022377705). We report this meta-analysis in compliance with the Preferred Reporting Items for Systematic Reviews and Meta-Analyses (PRISMA 2020) guidelines (Supplementary Table S1) (Page et al., 2021).

### 2.1 Search strategy

We searched PubMed, Cochrane Library, Embase, Chinese Science and Technology Periodicals (VIP) and China National Knowledge Infrastructure (CNKI) from their inception to 23 March 2023 without language restriction to obtain studies. Search terms were composed of medical subject headings (MeSH) and free words, and Boolean operators “AND,” “OR,” “NOT” were used to combine all search sets. Detailed search strategies are listed in Supplementary Table S2. We also screened clinicaltrials.gov and the reference lists of published reviews to identify additional relevant studies.

### 2.2 Eligibility criteria and study selection

Two qualified investigators (N.Y, W.D.W) independently evaluated the eligibility of the retrieved articles. Disagreements were settled through discussion with a third investigator (H.P.X). All eligible studies were checked by reading the abstract first, followed by reading the full-text. Additional articles were found by searching the references of the retrieved articles.

We included cohort studies and randomized controlled trials (RCTs) according to the following criteria: 1) atropine in at least one treatment arm and placebo/no intervention in another as the control; 2) participants were under 18 years old and had myopia (spherical equivalence, SE ≤ −0.5 diopters (D), under cycloplegia); 3) reported at least one outcome of myopia progression (i.e., refraction progression and elongation of AL) and/or side effects of atropine therapy; 4) the duration of follow-up was at least 1 year. We also designated 0.5% tropicamide as placebo because Shih et al. (1999) previous study found that 0.5% tropicamide had a similar effect on myopia progression to placebo. Similarly, single vision spectacle lenses were prespecified as a no intervention treatment. Studies published as case reports, comments, conference abstracts, editorials, letters, nonhuman studies, reviews and studies with duplicate data or without applicable data were excluded. The reasons for exclusion are shown in Supplementary Table S3.

### 2.3 Data extraction and quality assessment

A data extraction form included the first authors’ surname, year of publication, country, mean age, follow-up duration, treatment arm, sample size, baseline characteristics of the participants and endpoints of interest. Two investigators (H.P.X., W.D.W) independently retrieved the data and performed the quality and risk of bias assessment, and disagreements were resolved by the third investigator (Y.G.Y.). For any missing data, we emailed the corresponding authors at least twice or used GetData GraphDigitizer 2.24 to extract the data from the figures. The risk of bias of the selected RCTs was assessed by the recommendations of the Cochrane collaboration for RCTs (RoB 2) (Sterne et al., 2019), while the quality of the included cohort studies was assessed by the Newcastle‒Ottawa Quality Assessment Scale (NOS) (Wells et al., 2014). Articles with a NOS score ≥7 were considered to be high quality and those with a NOS score less than 7 would be excluded.

### 2.4 Primary and secondary outcomes

Primary outcomes were mean annual change in refraction (D/year) and mean annual change in AL (mm/year). They were all used to evaluate the efficacy of atropine.

Secondary outcomes included risk of rapid (>1.0 D/year)/slow (<0.5 D/year) myopia progression (Gan et al., 2021; Ha et al., 2022), progression in different treatment duration (D/year, mm/year), rebound effect (D/year, mm/year), progression in 3 years (D, mm), accommodation change (D), photopic pupil diameter change (mm), best-corrected visual acuity change (BCVA, logMAR/year), astigmatism change (D/year), anterior chamber depth change (ACD, mm/year), corneal curvature change (D/year), intraocular pressure change (IOP, mmHg/year), lens thickness change (LT, mm/year) and incidence of photophobia, blurred near vision and allergic reactions. Among them, the first four outcomes evaluated the efficacy of atropine, and the other secondary outcomes evaluated the safety of atropine.

### 2.5 Statistical analysis

Data analyses were performed using Review Manager 5.3 (www.reviewmanager.co.uk), STATA 12.0 (StataCorp, United States) and SPSS 26.0 (IBM United States) software. We computed the weighted mean difference (WMD) and 95% confidence interval (CI) for refraction changes (calculated as SE) and axial elongation in different doses of atropine arms and the control group, as well as the odds ratio (OR) for rapid/slow myopia progression and adverse effects. The effect sizes (ESs) were calculated using the Cohen d formula. Positive ESs for spherical equivalent and pupil dilation, as well as the mean difference, indicated that the atropine was superior to the control in terms of its effect on the increase in these outcomes, whereas the converse was true for AL and the change in accommodation amplitude (Fritz et al., 2012). An effect size would be defined as small at 0.20 or greater, medium at 0.50 or greater, or large at 0.80 or greater, which means the treatment effect was low, moderate, or strong, respectively (Cohen, 1977). The relationship between ESs from the meta-analysis and different concentrations of atropine was determined using linear, logarithmic, inverse, quadratic, cubic fits, *etc. R* ^2^, standardized residual errors and *p*-value were used to determine the model that best fits the data. At the same time, linear regression was used to determine whether there was a linear relationship between atropine dose and rebound effect, 3-year refraction and risk of adverse events, respectively. Heterogeneity was assessed using Cochran’s Q-test and *I* ^2^ statistics. If the heterogeneity was not statistically significant (*p* > 0.1, *I* ^2^ < 50.0%), a fixed-effects model was used; otherwise, a random-effects model was used. As much less studies focused on other doses than 0.01%, we could not get enough data on each specific concentration for meta-analysis, thus, subgroup analysis was performed by comparing the effects of different doses [low (0.005%–0.05%), moderate (0.1%–0.25%), high (0.5%–1%)] on myopic control. This stratification is based on the concentrations, customary name and mainstream trend that are the main focus of current studies (Yam et al., 2019; Jonas et al., 2021). The leave-one-out method was used for sensitivity analysis. Meta-regression analysis was used to assist in exploring the sources of heterogeneity. In addition, publication bias was first evaluated by visual inspection of funnel plots and then addressed by Egger’s and Begg’s tests. *p* < 0.05 was considered statistically significant for all analyses.

## 3 Results

**FIGURE 1:**
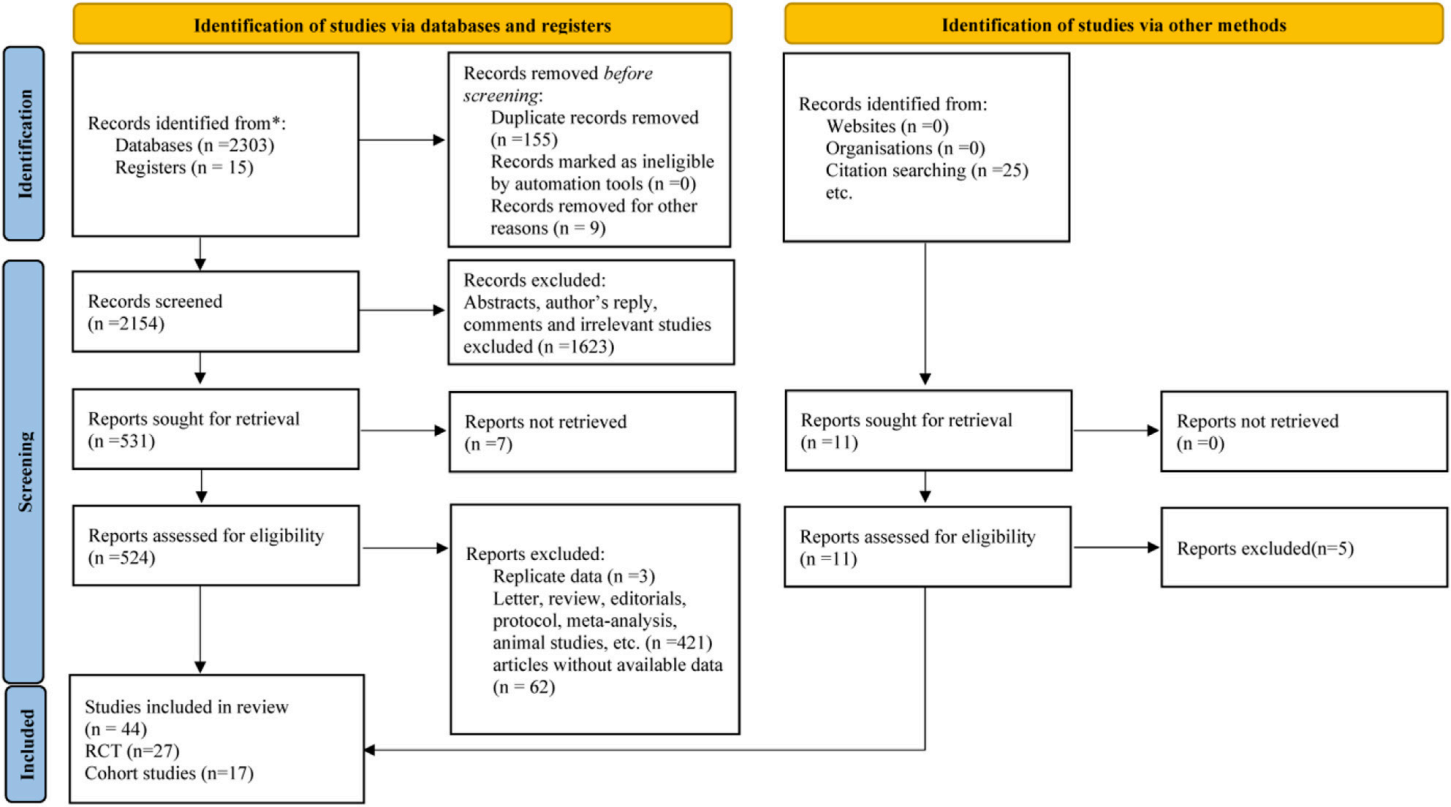
Flow chart of study selection. RCT, randomized controlled trial.

The search yielded a total of 2318 articles, of which 27 RCTs and 17 cohort studies were included in the final analysis (Figure 1). Baseline characteristics of the included studies were shown in Supplementary Table S4.

### 3.1 Risk of bias assessment

Two RCTs were assessed as high-risk articles mainly due to no or imperfect blinding (Supplementary Table S5). All the included cohort studies were assessed as high-quality studies (Supplementary Table S6). One cohort study was excluded due to its NOS score was lower than 7 (Erdinest et al., 2022).

### 3.2 Efficacy

#### 3.2.1 Effect of atropine on annual refraction change

**FIGURE 2:**
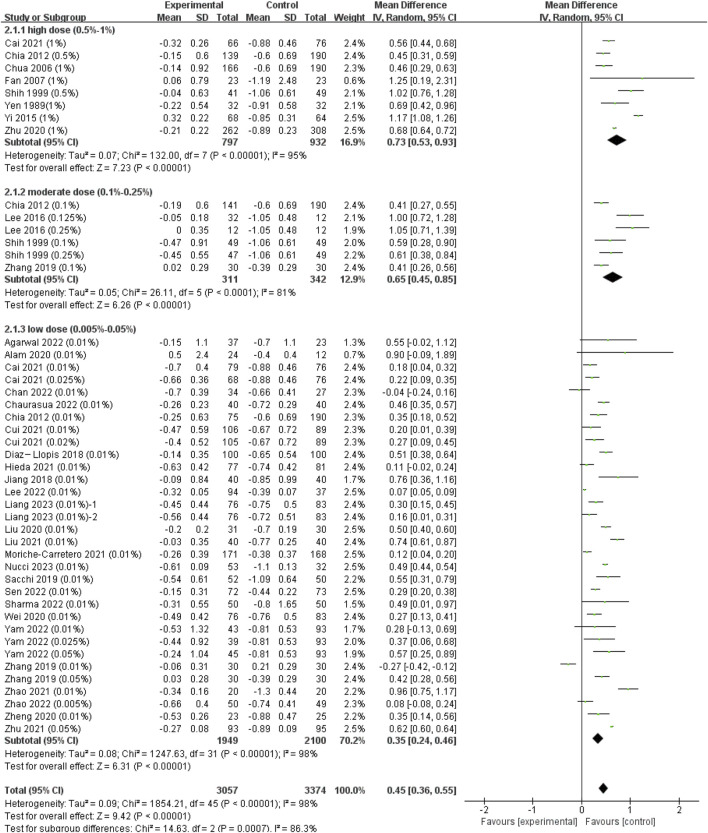
Effect of atropine on refraction change (D/year). CI, confidence interval; SD, standard deviation.

**FIGURE 3:**
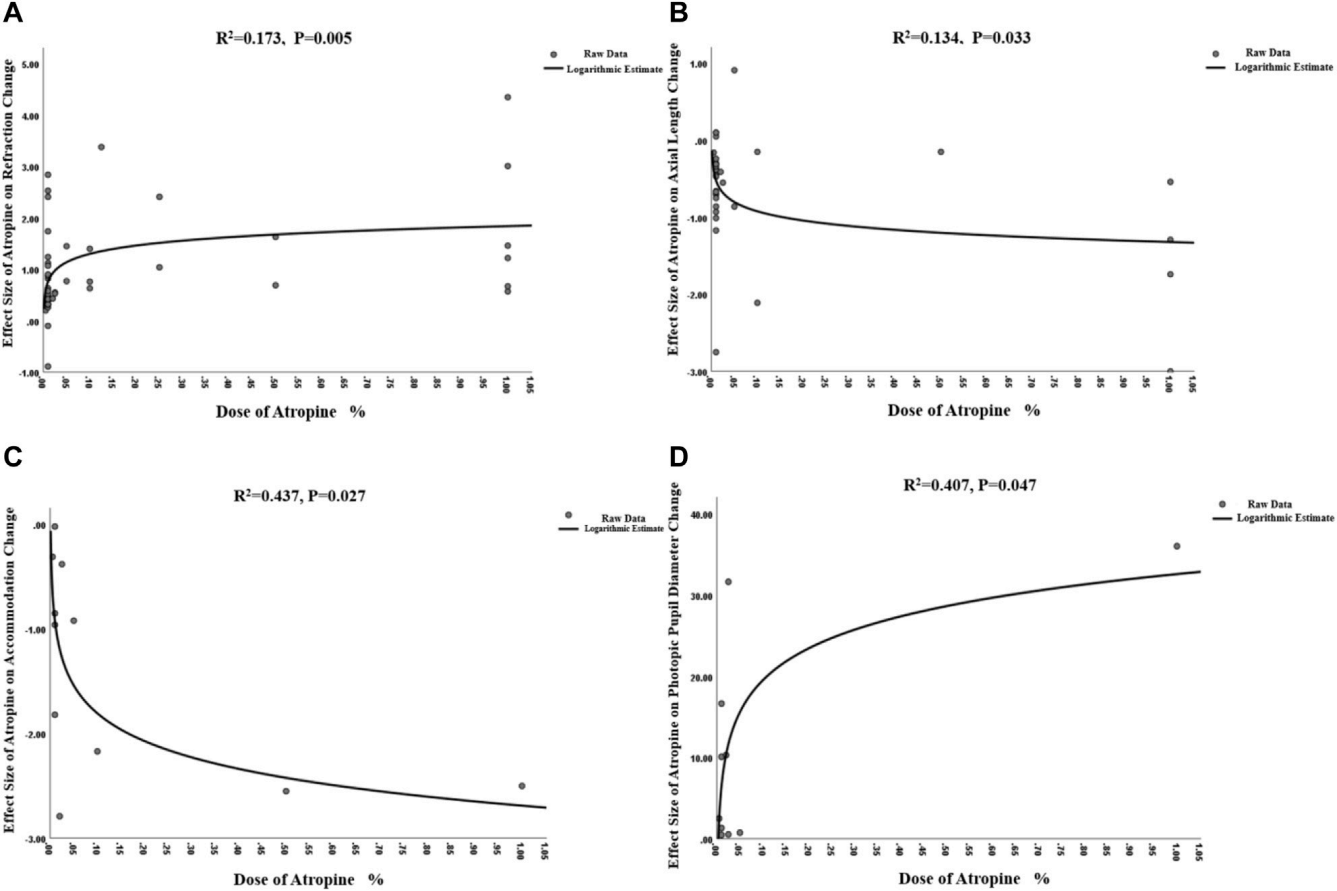
Non-linear dose-response relationship between atropine dose and refraction change, axial length change, accommodation change and photopic pupil diameter change. **(A)** Non-linear dose-response relationship between atropine dose and refraction change. **(B)** Non-linear dose-response relationship between atropine dose and axial length change. **(C)** Non-linear dose-response relationship between atropine dose and accommodation change. **(D)** Non-linear dose-response relationship between atropine dose and photopic pupil diameter change.

Since we did not find significant difference among RCT and cohort studies (*I* ^2^ = 0%, *p* = 0.51), we combined all the data to provide a larger sample in different doses (Supplementary Figure S1A). The pooled data showed significantly less progression in refraction for low-dose (WMD, 0.35 D per year; 95% CI, 0.24-0.46 D per year; *p* < 0.001), moderate-dose (WMD, 0.65 D per year; 95% CI, 0.45-0.85 D per year; *p* < 0.001), and high-dose (WMD, 0.73 D per year; 95% CI, 0.53-0.93 D per year; *p* < 0.001) atropine groups than control groups (Figure 2). And the subgroup difference was of statistical significance (χ^2^ = 14.63; *I* ^2^ = 86.3%; *p* < 0.001). The ESs of different doses of atropine groups all showed large treatment effects (Supplementary Figure S2A). In exploring the dose‒response relationship between atropine dose and ESs, we observed a significant linear correlation after excluding Zhu’s and Nucci’s studies which provided extreme findings (y = 1.08x + 0.86; *r* = 0.370, *R* ^2^ = 0.137; *p* = 0.013; Supplementary Figure S3A) (Zhu et al., 2021; Nucci et al., 2023). However, when we further exploring their nonlinear correlation, we found that the logarithmic equation has a better fitting effect (y = 0.23lnx+1.84, *R* ^2^ = 0.173, *p* = 0.005, Figure 3A). The curve is steep at lower atropine concentrations and seems to flatten from approximately 0.05%.

#### 3.2.2 Effect of atropine on annual axial length change

**FIGURE 4:**
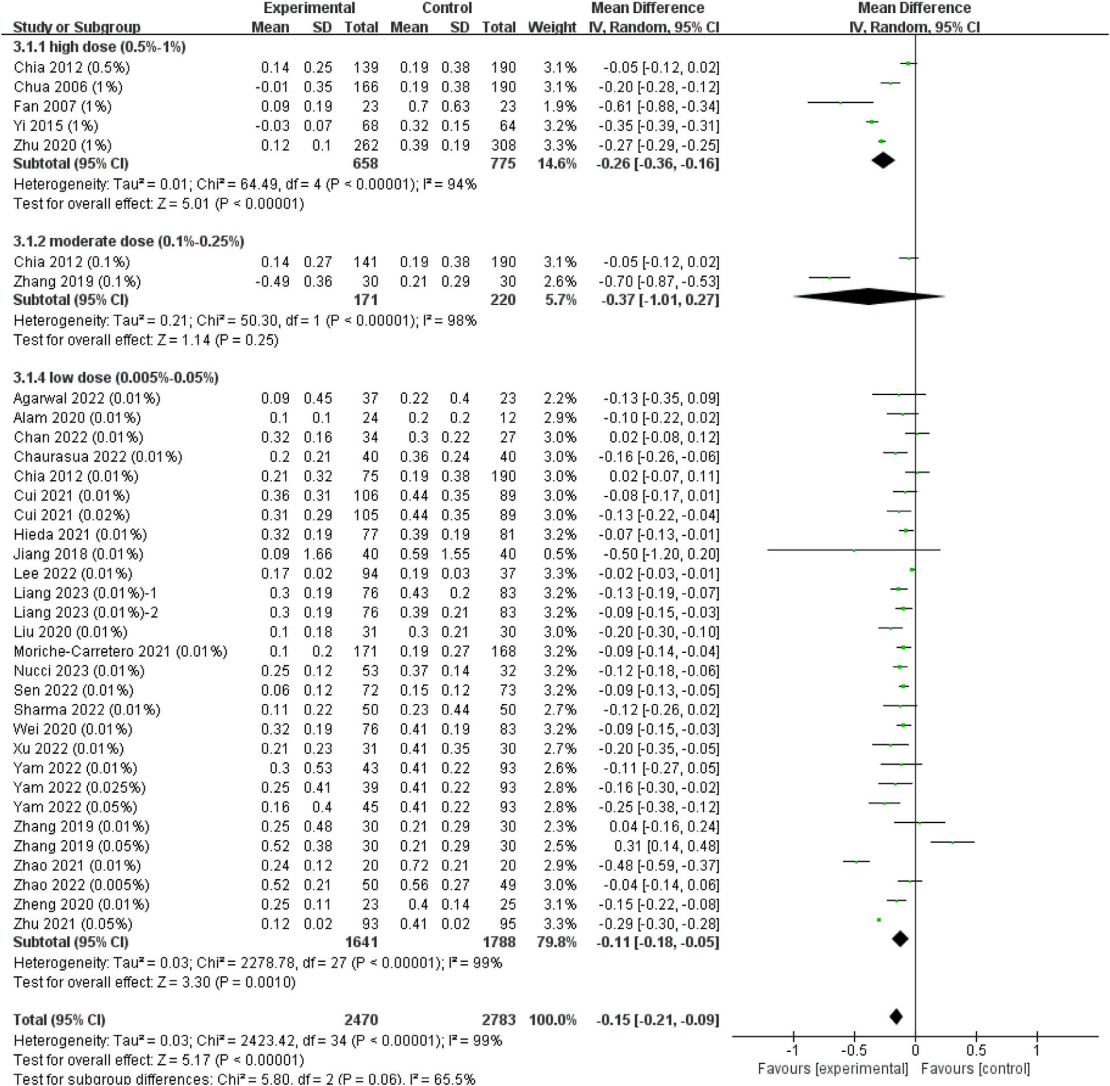
Effect of atropine on axial length change (mm/year). CI, confidence interval; SD, standard deviation.

The heterogeneity between different study types was not statistically significant (*I* ^2^ = 0%, *p* = 0.43) (Supplementary Figure S1B), so we finally combined the results of RCTs and cohort studies to expand the sample size. The analyses showed that the WMD in changes in AL between the atropine groups and control groups was −0.11 mm in low-dose group (95% CI, −0.18, −0.05; p = 0.001), −0.37 mm in moderate-dose group (95% CI, −1.01, 0.27; *p* = 0.25) and −0.26 mm (95% CI, −0.36, −0.16; p < 0.001) in high-dose group (Figure 4), though the difference among subgroups was not statistically significant (χ^2^ = 5.80; *I* ^2^ = 65.5%; *p* = 0.06). The ESs showed a large treatment effect for annual axial length change in subgroups with high dose and moderate dose, and a moderate treatment effect for low dose group (Supplementary Figure S2B). When examining the dose-response relationship, we observed a significant linear correlation (Supplementary Figure S3B, y = −1.01x−0.53, r = 0.426, *R* ^2^ = 0.181, *p* = 0.012) and a non-linear correlation (Figure 3B, y = −0.17lnx−1.32, *R* ^2^ = 0.134, *p* = 0.033) after excluding Zhu et al. (2021) study that provided extreme findings. The logarithmic curve is steep at lower atropine concentrations and seems to flatten from approximately 0.05%.

#### 3.2.3 Risk of rapid myopia progression (>1.0 D/year) and slow myopia progression (<0.5 D/year)

**FIGURE 5:**
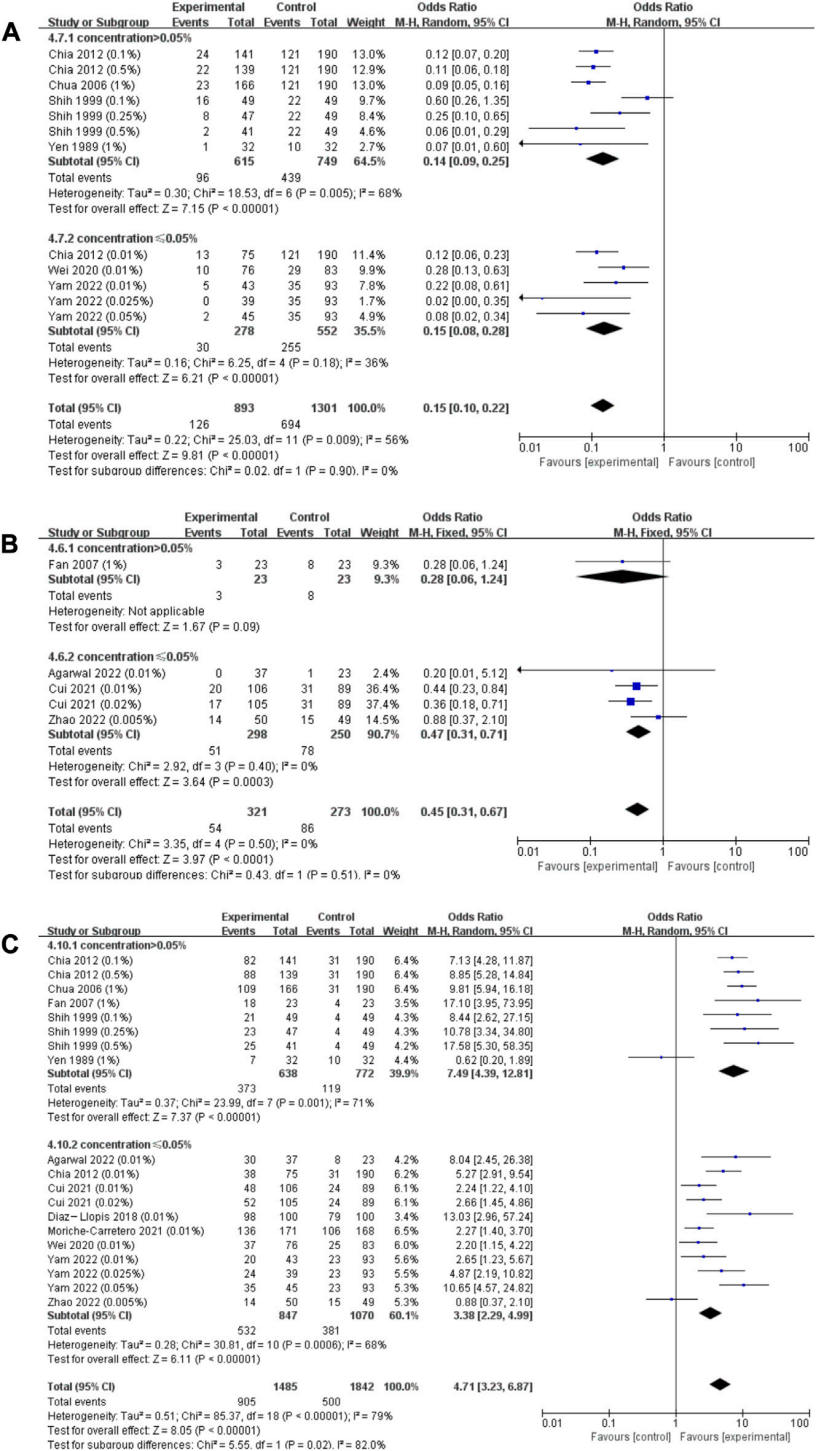
Effect of atropine on risk of rapid myopia progression (>1.0 D/year) and slow myopia progression (<0.5 D/year). **(A)** Effect of atropine on risk of rapid myopia progression (>1.0 D/year) (RCTs only). **(B)** Effect of atropine on risk of rapid myopia progression (>1.0 D/year) (cohort studies only). **(C)** Effect of atropine on risk of slow myopia progression (<0.5 D/year). CI, confidence interval; SD, standard deviation.

Since the heterogeneity between RCTs and case‒control studies was statistically significant (χ^2^ = 16.15; *I* ^2^ = 93.8%; *p* < 0.001; Supplementary Figure S4A), we respectively pooled the data of RCTs and cohort studies. According to the dose‒response curves mentioned above, the effect of atropine on annual refraction change and axial length change slowed down when its concentration was higher than 0.05%, we stratified the pooled data by the 0.05% concentration cutoff. Both pooled results showed that atropine of concentration higher than 0.05% resulted in relatively lower risk of rapid myopia progression, but the subgroup differences was not statistically significant (RCTs: concentration (conc) > 0.05%: OR = 0.14; 95% CI, 0.09–0.25; *p* < 0.001; conc ≤0.05%: OR = 0.15; 95% CI, 0.08–0.28; *p* < 0.001, χ^2^ = 0.02; *I* ^2^ = 0%; *p* = 0.90; Figure 5A; Cohort studies: conc >0.05%: OR = 0.28; 95% CI, 0.06–1.24; *p* = 0.09; conc ≤0.05%: OR = 0.47; 95% CI, 0.31–0.71; *p* < 0.001; χ^2^ = 0.43; *I* ^2^ = 0%; *p* = 0.51; Figure 5B).

Due to low heterogeneity among different research types (χ^2^ = 0.36; *I* ^2^ = 0%; *p* = 0.55; Supplementary Figure S4B), we combined data from these studies to evaluate the risk of slow myopia progression. We stratified the pooled values by a concentration of 0.05%, and the results showed concentration higher than 0.05% resulted in significantly higher risk of slow myopia progression (conc >0.05%: OR = 7.49; 95% CI, 4.39–12.81; *p* < 0.001; conc ≤0.05%: OR = 3.38; 95% CI, 2.29–4.99; *p* < 0.001) and the heterogeneity between subgroups was statistically significant (χ^2^ = 5.55; *I* ^2^ = 82.0%; *p* = 0.02; Figure 5C).

#### 3.2.4 The progression of refraction and axial length in different treatment duration

A total of 7 studies were included when we compared changes in refraction and AL in the first and second year of two consecutive years of treatment. We stratified the pooled data by the 0.05% concentration cutoff (Supplementary Figure S5). Results showed that there was no significant change in refraction in different treatment duration (*p* = 0.27; subgroup heterogeneity: *I* ^2^ = 0%, *p* = 0.53), but the subgroup with a concentration ≤0.05% showed a relatively stronger effect on slowing axial elongation in the second year (conc >0.05%: −0.09 mm, 95% CI, −0.16, −0.01, *p* = 0.03; conc ≤0.05%: 0.04 mm, 95% CI, 0.00–0.08, *p* = 0.04; subgroup difference: *I* ^2^ = 87.1%, *p* = 0.005).

#### 3.2.5 Rebound effect

Four studies took patients off medications for 1 year after 2 years of continuous treatment, and there were 8 treatment arms in total. The changes in refraction and AL in the third year were pooled and then stratified with a 0.05% cutoff (Supplementary Figure S6). The pooled results showed that there was a relatively stronger rebound effect in the higher dose group, although the heterogeneity between subgroups in the change in refraction was not statistically significant (*I* ^2^ = 4.8, *p* = 0.31). Therefore, we further explored the correlation between treatment dose and rebound effect of atropine effect on refraction change, and found a statistically significant correlation (*r* = 0.883, *p* = 0.004).

#### 3.2.6 Refraction and axial length progression in 3 years

A total of 4 studies consisting of 8 treatment arms examined the efficacy of myopia control in all 3 years (2 years of continuous treatment and 1 year washout stage). As mentioned above, we stratified the pooled values by a concentration of 0.05% and the results showed that both subgroups could significantly delayed refraction progression (conc ≤0.05%: −0.99 D, 95% CI, −1.36, −0.62; conc >0.05%: −1.33 D, 95% CI, −1.82, −0.83; subgroup heterogeneity: *I* ^2^ = 12.9%, *p* = 0.28) and axial elongation (concentration ≤0.05%: 0.38 mm, 95% CI, 0.25–0.51; concentration >0.05%: 0.50 mm, 95% CI, 0.09–0.92; subgroup heterogeneity: *I* ^2^ = 0%, *p* = 0.59) but the subgroup differences were not statistically significant (Supplementary Figure S7).

### 3.3 Safety

#### 3.3.1 Effect of atropine on accommodation changes

**FIGURE 6:**
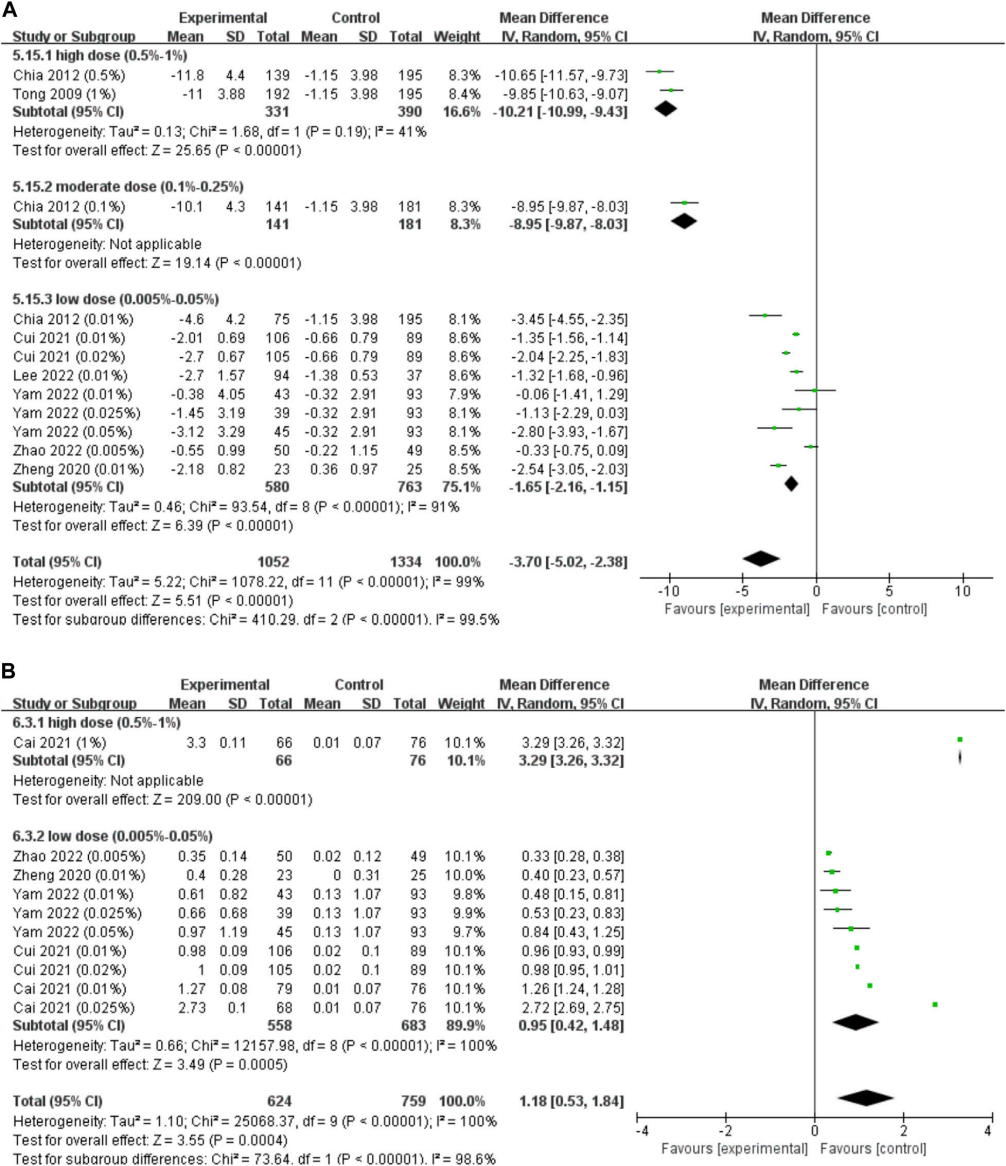
Effect of atropine on accommodation change and photopic pupil diameter change. **(A)** Effect of atropine on accommodation change (D). **(B)** Effect of atropine on photopic pupil diameter change (mm). CI, confidence interval; SD, standard deviation.

Only 7 studies reported changes in accommodation. As some parameters, such as the amplitude of accommodation and photopic pupil diameter, changed the most in the first few months and these changes almost lasted over the whole course of successive treatments (Tong et al., 2009; Yam et al., 2019), we pooled these data regardless of the different follow-up durations (Figure 6A). We found that atropine resulted in a significant reduction in the amplitude of accommodation (WMD, −3.70 D; 95% CI, −5.02, −2.38; *p* < 0.001), and higher concentrations resulted in more reduction in accommodation (*I* ^2^ = 99.5%, *p* < 0.001).

When investigating the dose‒response relationship, we found a significant logarithmic correlation (not a linear correlation, *r* = 0.546, *p* = 0.082) between different concentrations and its ESs on accommodation amplitude changes after excluding Zheng et al. (2020) study which provided extreme findings (y = −0.38lnx-2.69, *R* ^2^ = 0.437, *p* = 0.027; Figure 3C). The curve was steep at lower atropine concentrations and became flat from approximately 0.1%. Therefore, we respectively pooled the changes in accommodation under the treatment of atropine of concentrations <0.1% and ≥0.1% (Table 1). We found that atropine with a concentration <0.1% would cause a decrease in accommodation amplitude less than 4 D, while atropine with a concentration higher than 0.1% would possibly decrease the accommodation as high as 10 D.

**TABLE 1:** Subgroup analysis for amplitude of accommodation and photopic pupil diameter under treatment of atropine.

Outcome parameter	Follow-up	Subgroup	Mean difference	95% CI
Amplitude of accommodation (D)	2 years	concentration≥0.1%	−10.93	(−16.96, −4.90)
		concentration<0.1%	−2.36	(−3.22, −1.49)
	1 year	concentration≥0.1%	−11.79	(−16.78, −6.80)
		concentration<0.1%	−1.59	(−2.64, −0.54)
Photopic pupil diameter (mm)	2 years	Concentration≥0.1%	3.29	(3.07, 3.50)
		Concentration<0.1%	1.28	(0.66, 1.90)
	1 year	Concentration≥0.1%	2.71	(1.34, 4.07)
		Concentration<0.1%	0.43	(0.21, 0.65)

*CI, confidence interval.

#### 3.3.2 Effect of atropine on pupil diameter change

Five studies reported changes in photopic pupil diameter, only 1 of which reported changes of mesopic pupil diameter. As photophobia is usually present in bright environments, we only pooled the data of photopic pupil diameter change (Figure 6B). Regardless of the duration of follow-up, we found that the photopic pupil diameter was significantly larger in the atropine groups (WMD, 1.18 mm; 95% CI, 0.53-1.84; *p* < 0.001), and higher concentrations resulted in larger photopic pupil dilations (*I* ^2^ = 98.6%, *p* < 0.001).

The atropine dose and its effect on photopic pupil diameter change were simultaneously fitted linearly and nonlinearly. Both the linear correlation (y = 28.23x+7.75, r = 0.663, *R* ^2^ = 0.439, *p* = 0.037; Supplementary Figure S3C) and the nonlinear correlation were of statistical significance (y = 5.67 lnx+32.60, *R* ^2^ = 0.407, *p* = 0.047; Figure 3D). The logarithmic curve was steep at lower atropine concentrations and became flat from approximately 0.1%. Therefore, we respectively pooled the changes in photopic pupil diameter under the treatment of atropine of concentrations <0.1% and ≥0.1% and found that atropine with a concentration <0.1% would cause photopic pupil dilation of no more than 3 mm (Table 1).

#### 3.3.3 Effect of atropine on BCVA, astigmatism, anterior segment, IOP and LT

Results of the meta-analysis showed that both high dose and moderate dose atropine could significantly decrease distant BCVA (high dose: WMD, −0.38; 95% CI, −0.39, −0.37; p < 0.001; moderate dose: WMD, −0.08; 95% CI, −0.10, −0.06; *p* < 0.001; Supplementary Figure S8A), but they only included one trial. Similarly, high dose atropine could significantly increase corneal astigmatism (high dose: WMD, 0.03 D; 95% CI, 0.00-0.06; *p* = 0.04; Supplementary Figure S8B), but it only included one trial. No statistically significant differences were found between the atropine and control groups in IOP, anterior chamber depth (ACD) and corneal curvature (Supplementary Figure S8). Only Lee et al. reported changes in lens thickness (LT), but its results showed that atropine had no significant effect on the change in LT (Lee et al., 2022).

#### 3.3.4 Adverse events

Pooled results suggested that atropine can significantly increase the risk of photophobia (OR = 17.12; 95% CI, 6.34–46.23; *p* < 0.001), blurred near vision (OR = 16.40; 95% CI, 8.62–31.20; *p* < 0.001) and allergic reactions (OR = 4.13; 95% CI, 2.49–6.84; *p* < 0.001) (Supplementary Figure S9). Moreover, the correlation between the incidence of photophobia and dose of atropine was statistically significant (*r* = 0.477, *p* = 0.029).

### 3.4 Sensitivity analysis, meta-regression and publication bias

Only the sensitivity analysis for corneal astigmatism suggested that Chia 2009 might be the main source of heterogeneity (Chia et al., 2009). However, the data remained unchanged after the trim-and-fill method, which indicated that the study had little impact on heterogeneity. Therefore, the above results indicate that the original meta results are robust. Meta-regression analysis was performed to further explore the potential sources of heterogeneity for changes in refraction and AL, but the results did not indicate those parameters as the source of heterogeneity (Supplementary Figure S7).

Comprehensively considering the results of the funnel plots (Supplementary Figure S10), Begg’s and Egger’s tests, there might be some publication bias for the results of corneal astigmatism, photophobia and allergic reactions, however, trim-and-fill method suggested that these publication bias did not affect the robustness of these outcomes.

## 4 Discussion

### 4.1 Myopia control effect of atropine

In this meta-analysis, we combined the results from 27 RCTs and 17 cohort studies, and confirmed that there was significantly less myopia progression (WMD = 0.45 D/year; high dose: 0.73 D/year; moderate dose: 0.65 D/year, low dose: 0.35 D/year) and slower axial elongation (WMD = −0.15 mm/year; high dose: −0.26 mm/year; moderate dose: −0.37 mm/year, low dose: −0.11 mm/year) in the atropine group than in the control group. These results are similar to two previously published meta-analyses (Gong et al., 2017; Gan et al., 2021). Moreover, we observed linear and nonlinear dose−response correlations, suggesting that as the concentration increases, the ESs of atropine on retarding refraction and axial length progression increases. In addition, the logarithmic curves showed that, from a concentration of approximately 0.05%, the curve became flat. When it is above 0.05%, the ESs for retarding refraction and axial length progression do not increase dramatically with increasing concentration. This indicated that there may be a saturation point for atropine in the control of myopia.

Atropine had a significantly lower OR in children with rapid myopia progression and a significantly higher OR in children with slow myopia progression, which was consistent with the results of previous meta-analyses (Li et al., 2014; Gan et al., 2021; Ha et al., 2022). What’s more, our results suggested that atropine with concentrations higher than 0.05% were more effective in reducing the occurrence of rapid myopia progression and resulting higher probability of slow myopia progression.

### 4.2 Practical use of atropine in clinical work

#### 4.2.1 Feasibility of long-term clinical use

A full and detailed understanding of the specific control effect of atropine on myopia progression in different treatment periods and the rebound effect of different concentrations after withdrawal have important guiding significance for its clinical use. Our results showed that atropine of concentrations ≤0.05% showed relatively better efficacy in the second year with less refraction progression (WMD = −0.06 D, 95% CI, −0.15, 0.04, *p* = 0.24) and significantly less axial elongation (WMD = 0.04 mm, 95% CI, 0.00–0.08, *p* = 0.04), which indirectly supported the conclusions of the Gan et al.'s and ATOM2 studies (Chia et al., 2016; Gan et al., 2021). Meanwhile, high-dose atropine was relatively less effective in the second year. Although Chen et al. had explored the rebound effect of different doses previously, only two studies were pooled, and they were all from the same trial (ATOM) (Chen and Yao, 2021). Thus, we reexamined it and found a linear correlation—the higher the dose, the stronger the rebound effect, which is consistent with ATOM2 (Chia et al., 2014; Chia et al., 2016). Therefore, these results suggest that low dose atropine (0.005%–0.05%) show better efficacy in long-term myopia control.

ATOM2 reported that the 0.01% atropine treatment group had the least progression of myopia after the whole 3 years of follow-up (we named it the “three-year regimen”, including 2 years of continuous treatment and 1 year of cessation) (Chia et al., 2016), while our meta-analysis did not find a significant difference between the low-dose (0.005%–0.05%) and higher doses of atropine groups (meta-analysis of each concentration subgroup could not be performed due to lack of sufficient trials). However, due to the lack of long-term follow-up of the control groups in ATOM2 (Chia et al., 2012) and the switch of the control arm to treatment groups in the second year in LAMP (Yam et al., 2022), pooled results of the rebound effect and the control effect for the whole 3 years were based on the results of the treatment arms, which lack control groups for comparison, further research is needed to explore these issues.

#### 4.2.2 Appropriate changes of accommodation and pupil diameter

Except for the efficacy parameters, the amplitude of accommodation and pupil size are also important for the selection of atropine treatment concentration (Fang et al., 2010; Chia et al., 2012; Clark and Clark, 2015). Recent meta-analyses have analyzed these two outcome parameters (Chen and Yao, 2021; Gan et al., 2021; Tran et al., 2021; Ha et al., 2022), but the results were inconsistent. One study only pooled the data of the treatment arm (Gan et al., 2021), another study found no difference in photopic pupil diameter between the low-dose group and the control group (Ha et al., 2022), while others did not (Chen and Yao, 2021; Tran et al., 2021). Considering the recently released of the phase three of LAMP and the results of a study with a lower concentration of atropine (0.005%) reported these two parameters (Yam et al., 2022; Zhao et al., 2022), we reanalyzed them and further explored the nonlinear dose‒response correlation. Finally, we found that atropine could significantly reduce the amplitude of accommodation and increase the photopic pupil diameter. By nonlinear fitting, we found a significant logarithmic correlation in which the curve flattened from a concentration of approximately 0.1%, above which the reduction in accommodation amplitude and the increase in photopic pupil diameter became less pronounced. These were similar to the results of Tran et al. (2021). Furthermore, we pooled the changes in photopic pupil diameter and amplitude of accommodation for concentrations <0.1% and ≥0.1% respectively. We found that the increase in photopic pupil diameter did not exceed 3 mm when the dose was less than 0.1%. A concentration higher than 0.1% would cause a decrease in the amplitude of accommodation of more than 10 D, leaving the residual accommodation not exceeding 5D. Previous, some scholars supported the views of Cooper et al. that as long as the subjects have 5 D of accommodation and less than 3 mm of dilation, they will be able to perform their daily visual tasks without symptoms (Cooper et al., 2013). Of note, the concentrations included in the concentration <0.1% subgroup were all ≤0.05% (Chia et al., 2012; Yam et al., 2019; Fu et al., 2020; Yam et al., 2020; Zheng et al., 2020; Cai et al., 2021; Cui et al., 2021; Lee et al., 2022; Zhao et al., 2022). Therefore, based on the results of the current studies, we think that atropine concentrations ≤0.05% are the most suitable for clinical practical application from the perspective of changes in accommodation and pupil diameter. Given the small number of studies that can be included thus far, perhaps more research focusing on atropine at concentrations no more than 0.05% in the future can better address this issue.

#### 4.2.3 Other clinical safety parameters in atropine treatment

When exploring the effects of different doses of atropine on annual changes of BCVA, corneal astigmatism, IOP, and the anterior segment (corneal curvature, ACD and LT), we did not obtain a statistically significant result. We also pooled the first-year changes in these parameters, but the results differ little from results shown above, hence, they were not shown in this article.

The use of atropine eye drops will lead to mydriasis and accommodative paralysis, thus inducing photophobia and blurred near vision (McBrien et al., 2013; Upadhyay and Beuerman, 2020), and it can also cause local allergy, and other adverse events (Chia et al., 2012). Therefore, the safety of atropine eye drops in controlling the progression of myopia in children has always been the focus of clinical research. Previously, several studies have quantified the adverse events caused by atropine in the process of retarding myopia progression through meta-analysis (Gong et al., 2017; Gan et al., 2021; Ha et al., 2022), and their results all indicated that higher concentrations of atropine resulted in more adverse events, as mentioned above. Our study also demonstrated that atropine could significantly increase the risk of these major side effects, especially photophobia, which occurred more frequently as the treatment dose increased. Even so, photophobia and blurred near vision mostly appeared in the early stage of the treatment, the symptoms gradually relieved or disappeared with prolonged use, and photophobia could be well tolerated by the use of photochromic glasses in most patients (Yam et al., 2019; Fu et al., 2020). Allergic reactions included allergic conjunctivitis, itching of eye, eye swelling and redness and irritation, *etc.* However, the allergic reactions generally alleviated within 24 h after discontinuation of atropine eye drops and disappeared within a week (Kothari et al., 2018). Other adverse events included glare, burning of eyes, chalazion, and some systemic adverse events, such as facial flushing, dry mouth, dry skin, and irregular heart rate; some patients even had a severe adverse event requiring hospitalization (Tong et al., 2009; Chia et al., 2012; Larkin et al., 2019; Yam et al., 2019). Since these mostly happened side effects are associated with higher doses, lower doses of atropine are relatively safe.

### 4.3 Limitations and advantages of this study

This study has some limitations. First, although strict inclusion and exclusion criteria were established in this meta-analysis, heterogeneity remained high after the use of subgroup analysis. Second, due to insufficient data for some concentrations, different doses of atropine were combined in this meta-analysis, and the follow-up period varied significantly between trials. These might be the source of heterogeneity. Third, most of the assessed studies were conducted among Asians, so the pooled results should be interpreted in other races with caution. Finally, in spite of the sufficient included studies, only a few studies reported outcomes in terms of anterior segment, BCVA, IOP, astigmatism, amplitude of accommodation, pupil diameter and rebound effect, which is of great significance for judging the safety and compliance of atropine.

Despite these limitations, there are some advantages in our study: 1) Compared with previous systematic reviews, the effects of atropine on other outcomes (especially some parameters of the anterior segment of the eye, such as corneal curvature and anterior chamber depth) were further evaluated to more comprehensively explore the effects of different doses of atropine on myopia control. 2) The nonlinear dose‒response relationship between different atropine concentrations and refraction change, axial elongation, accommodation decrease, and photopic pupil enlargement were explored. These results may help us narrow the range of appropriate concentrations for clinical practice in the future. 3) Unlike the previous meta-analysis (Chen and Yao, 2021), we excluded the atropine combined with orthokeratology study because we could not exclude the possible complementation and combined effect. 4) Our study is the first systematic review to comprehensively analyze the overall effect of myopia control during 3 years of follow-up. 5) Our inclusion criteria were more stringent than those of previous meta-analyses. For example, we strictly restricted our study inclusion criteria to include only trials with baseline and follow-up refractive measurements under cycloplegia (Wang et al., 2017; Saxena et al., 2021), and excluded trials that lacked specific descriptions on the refractive range of the enrolled population or that the enrolled population had a refraction range beyond myopia (Han et al., 2019; Larkin et al., 2019).

## 5 Conclusion

Our meta-analysis showed that the higher the concentration of atropine, the slower progression of myopia, but also the greater the rebound effect. However, the overall myopia progression after 2 years of continuous treatment and 1 year of cessation is similar. There is no sufficient evidence to prove that the use of atropine would lead to changes in IOP, astigmatism, distant BCVA and other parameters of the anterior segment of the eye, especially when the concentration is lower than 0.05%. Major side effects of atropine treatment were photophobia, blurred near vision and allergic reactions, which were dose-dependent, but all were short-term effects. According to the trend of nonlinear dose-response curve and clinical experience, some major side effects (like decline in accommodation and increase in photopic pupil diameter that would lead to photophobia and blurred near vision) of atropine concentration ≤0.05% are tolerable while the myopia control effect is dose-dependent, therefore, maybe concentration of 0.05% is the best option to control myopia progression at present. Further trials of a range of gradients around this concentration should be carried out to help us quickly identify the concentration that can both effectively control myopia and minimize side effects, which it is also the optimal concentration that should be applied in the clinic.

## Data Availability

The original contributions presented in the study are included in the article/Supplementary Material, further inquiries can be directed to the corresponding author.
