# Supplementary material for: Comparison of the efficacy and safety of different doses of atropine for myopic control in children: a meta-analysis
Source: Front Pharmacol. 2023 Sep 11;14:1227787. doi: 10.3389/fphar.2023.1227787 (PMC10520549; doi:10.3389/fphar.2023.1227787)
Supplement: Supplementary file 1 [file DataSheet1.docx]

Supplementary Material

Comparison of the Efficacy and Safety of Different Doses of Atropine

for Myopic Control in Children: A meta-analysis

**Peixian Hou, Dawen Wu, Yan Nie, Hong Wei, Longqian Liu, Guoyuan Yang^*^**

*** Correspondence:** Guoyuan Yang: 31981077@qq.com

# Supplementary Figures and Tables

## Supplementary Figures

**(A)**

**
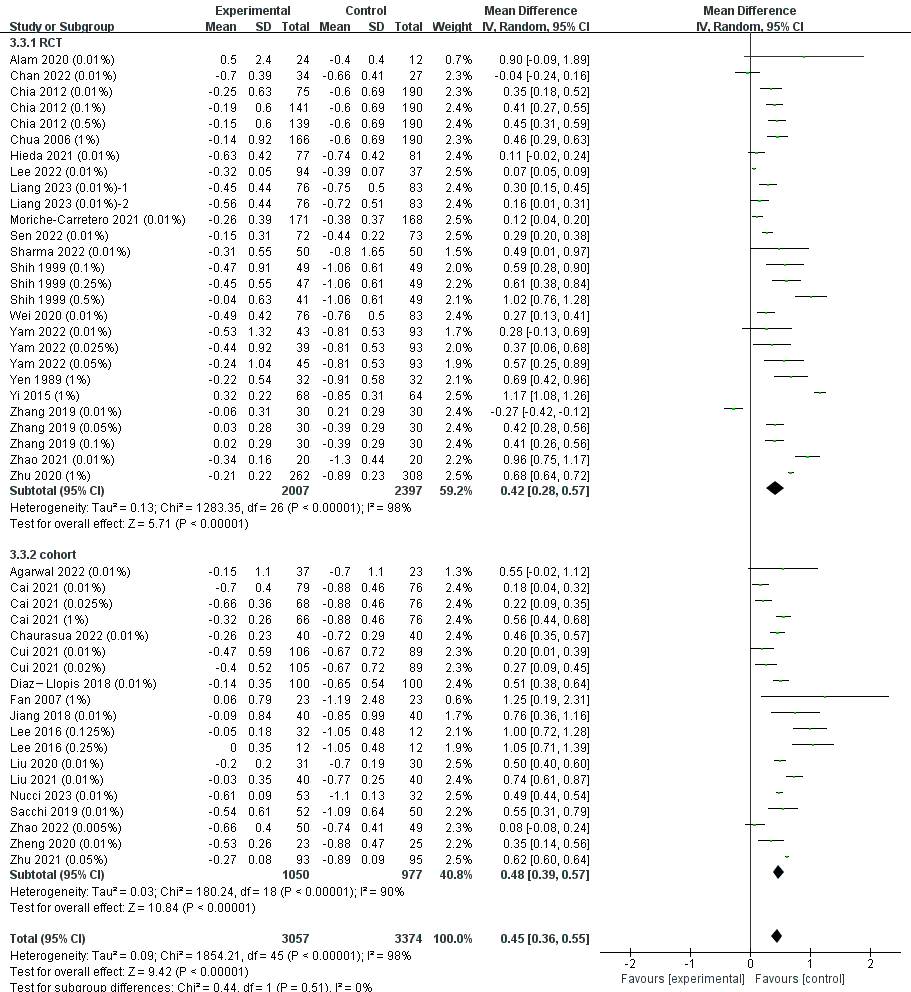
**

**(B)**

**
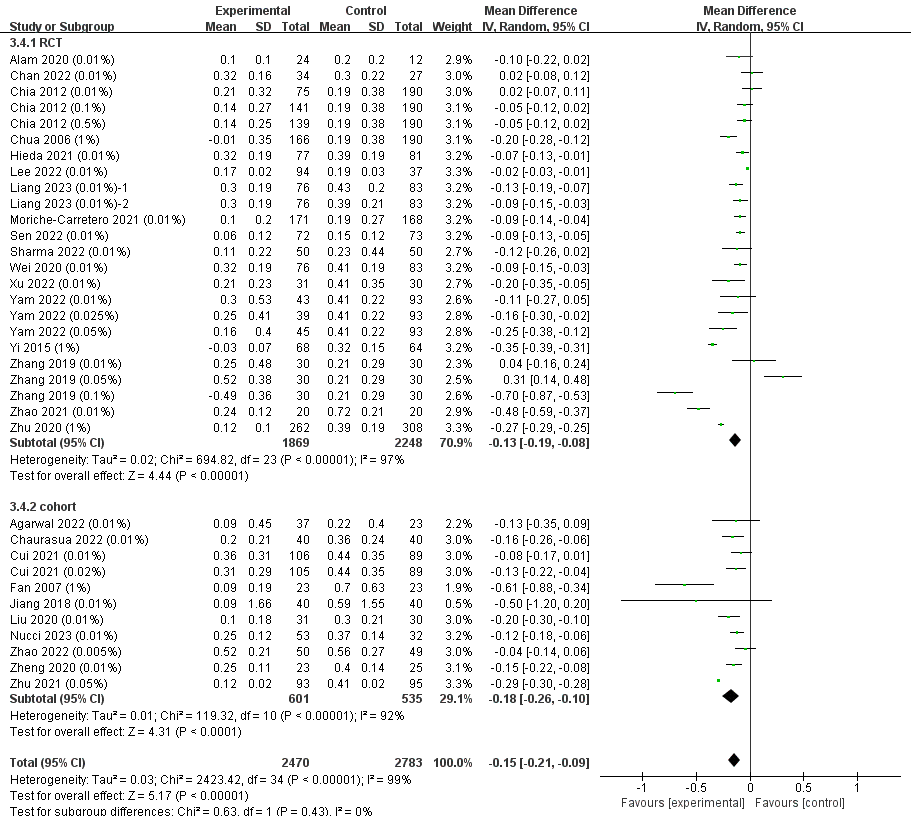
**

**Supplementary Figure 1.** **Effect of atropine on annual refraction change and axial length change (Stratified by study types).** **A,** Effect of atropine on annual refraction change (Stratified by study types) (D/year). **B,** Effect of atropine on annual axial length change (Stratified by study types) (mm/year). CI= confidence interval; SD= standard deviation.

**(A)**

**
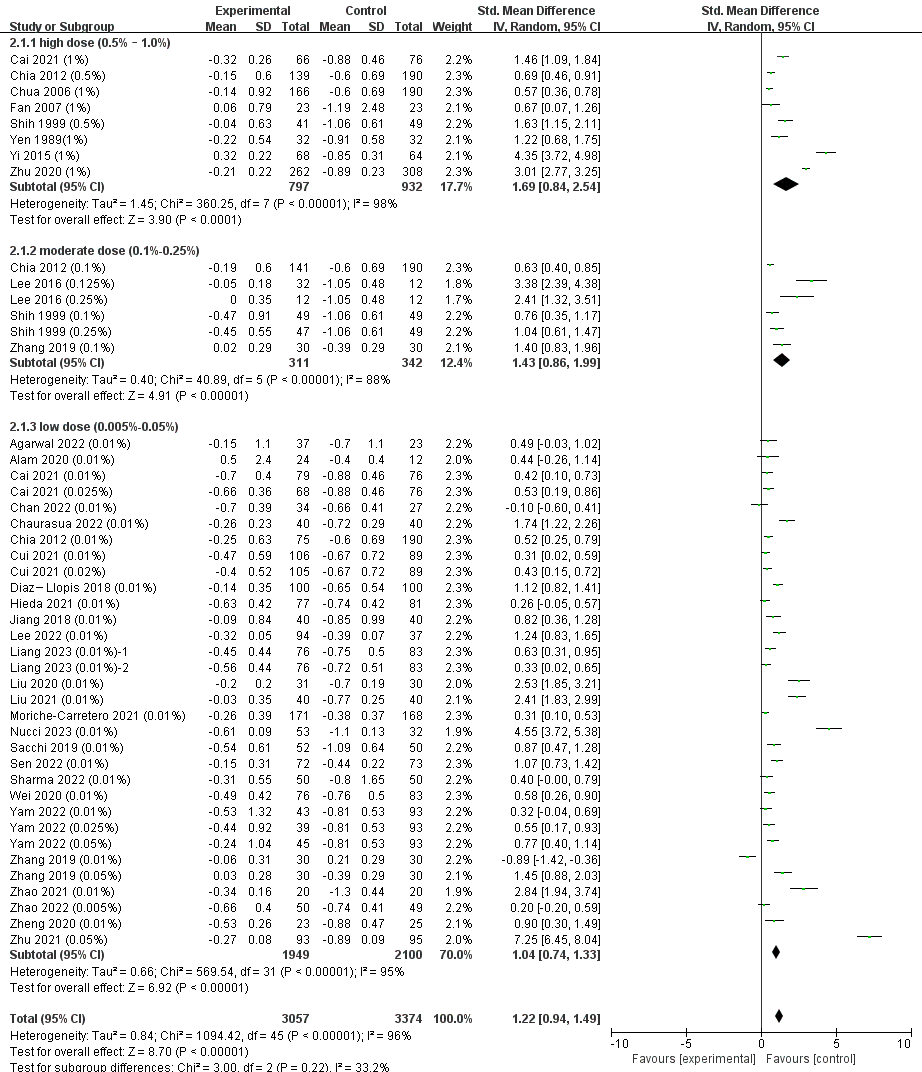
**

**(B)**

**
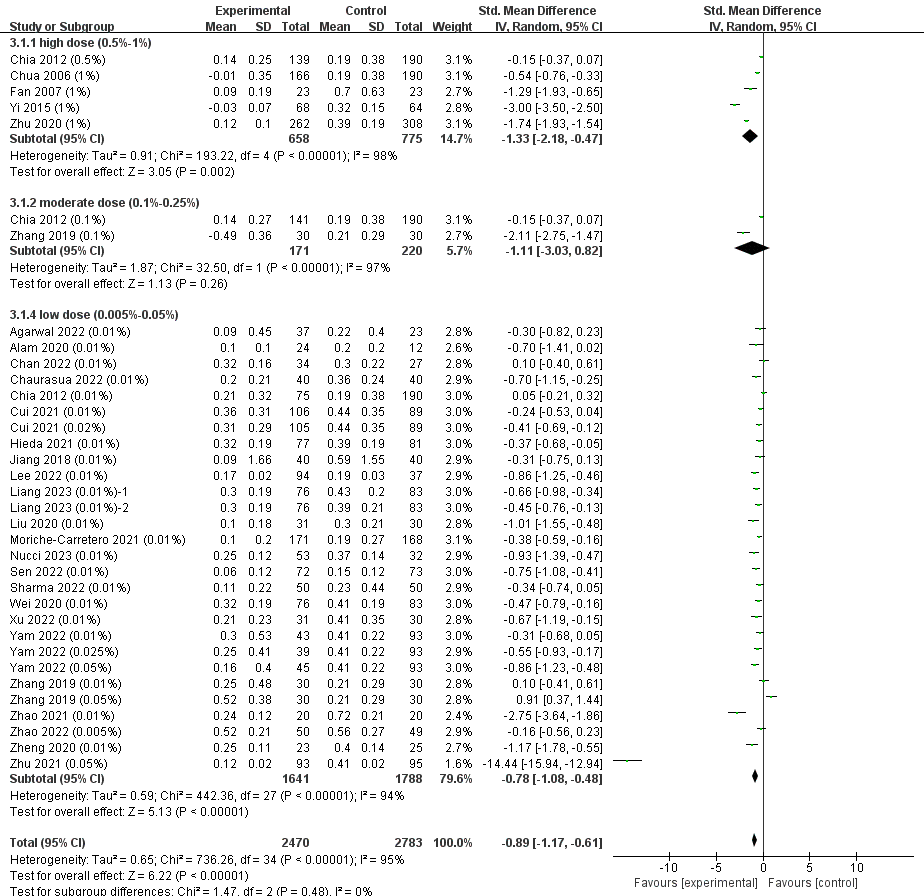
**

**Supplementary Figure 2.** **Effect size of atropine on annual refraction change and axial length change (Stratified by different doses).** **A,** Effect size of atropine on annual refraction change (Stratified by different doses) (D/year). **B,** Effect size of atropine on annual axial length change (Stratified by different doses) (mm/year). CI= confidence interval; SD= standard deviation.

**(A)**

**
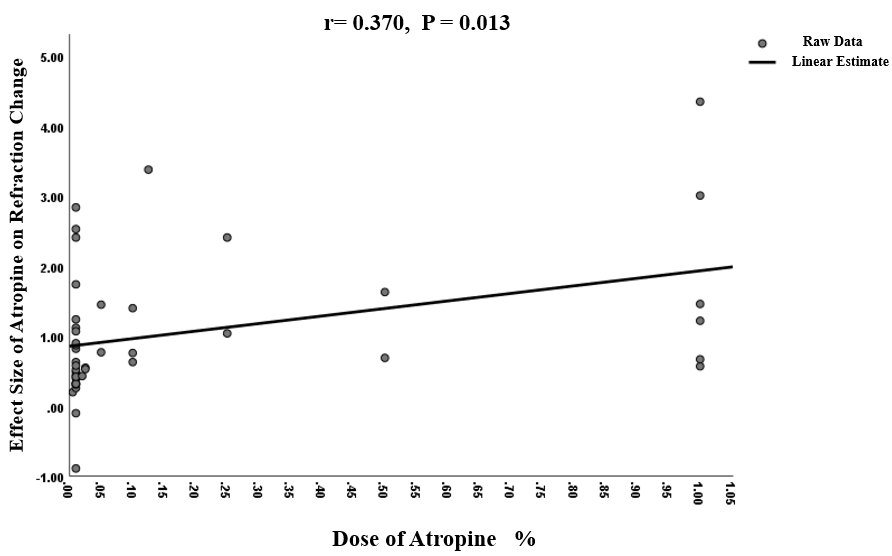
**

**(B)**

**
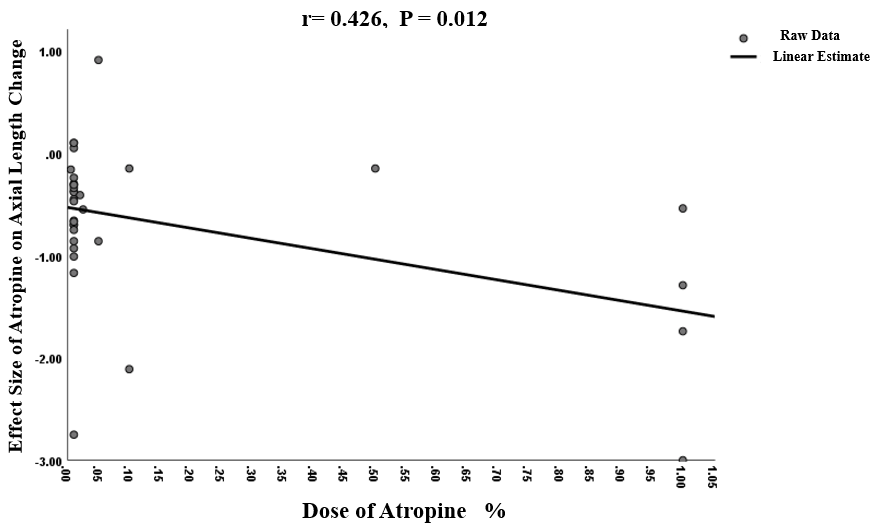
**

**(C)**


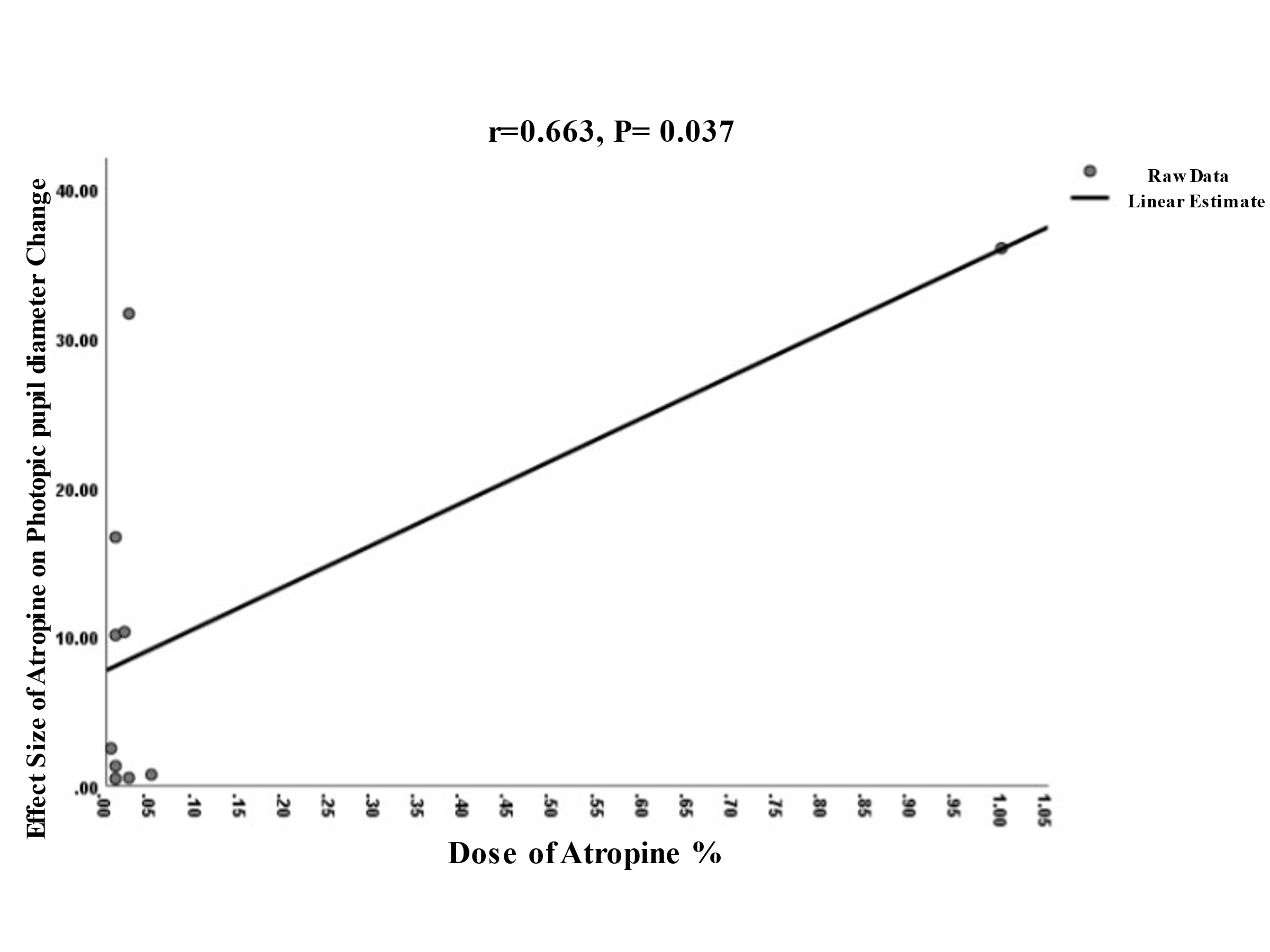


**Supplementary Figure 3.** **Linear dose-response relationship between atropine dose and refraction change, axial length change, photopic pupil diameter change. A,** Linear dose-response relationship between atropine dose and refraction change. **B,** Linear dose-response relationship between atropine dose and axial length change. **C,** Linear dose-response relationship between atropine dose and photopic pupil diameter change.

**(A)**

**
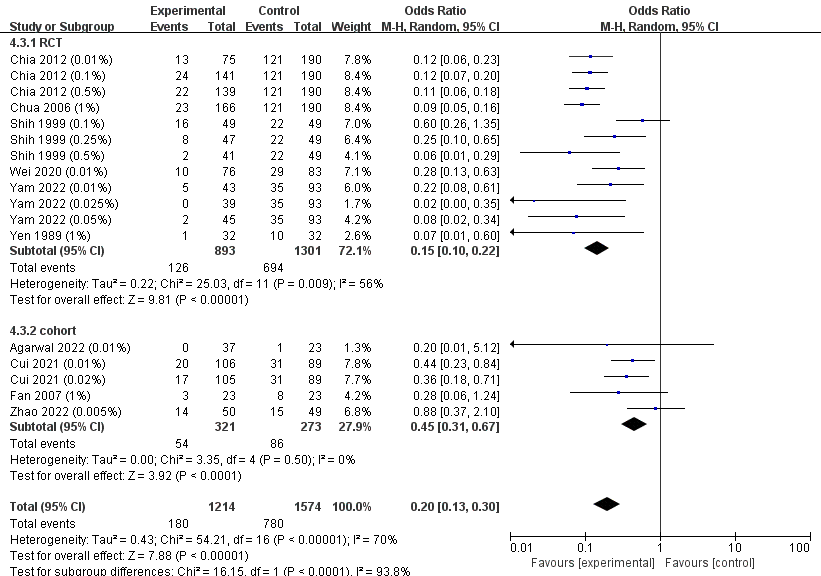
**

**(B)**

**
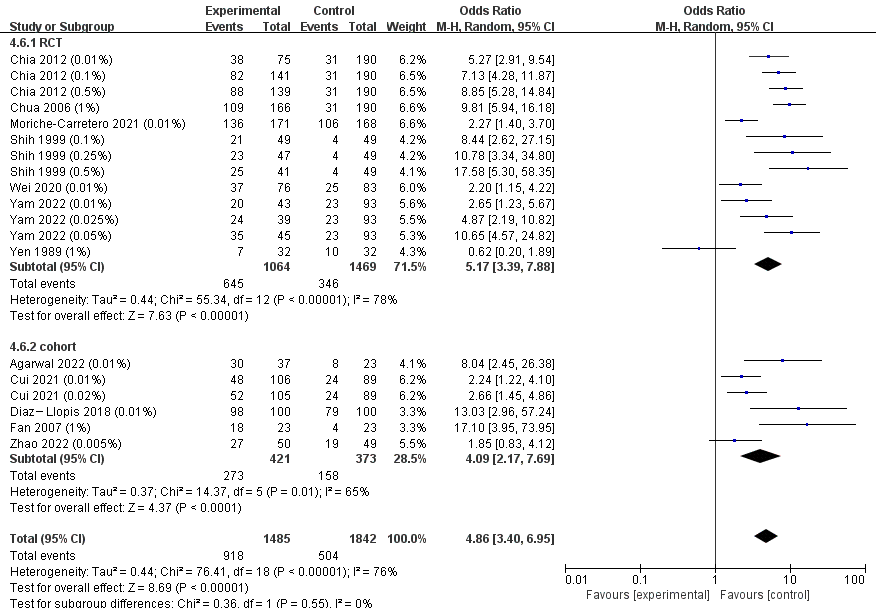
**

**Supplementary Figure 4.** **Effect of atropine on risk of rapid myopia progression (****>1.0 D/ year) and slow myopia progression (<0.5 D/ year) (Stratified by study types). A,** Effect of atropine on risk of rapid myopia progression (>1.0 D/ year). **B,** Effect of atropine on risk of slow myopia progression (<0.5 D/ year). CI= confidence interval; SD= standard deviation.

**(A)**

**
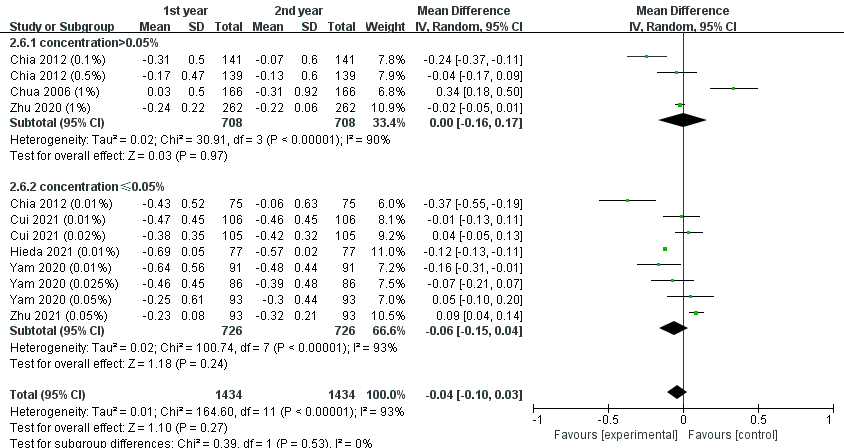
**

**(B)**

**
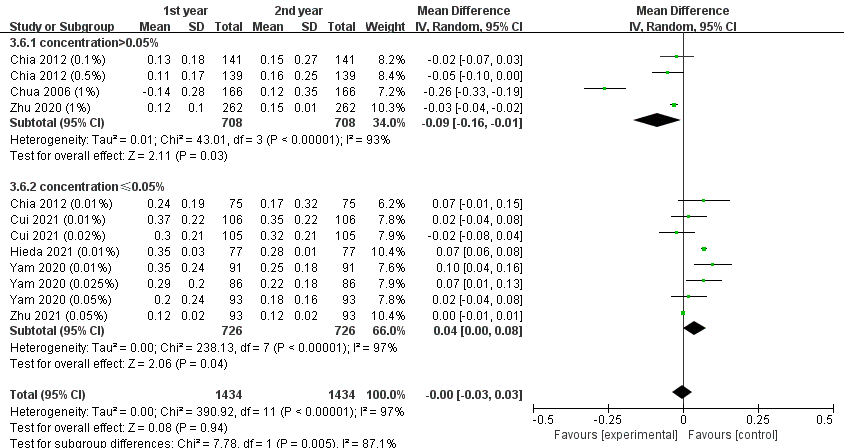
**

**Supplementary Figure 5.** **Comparison of different periods of refraction progression and axial elongation. A,** Effects of atropine on refraction changes in the first and second year of treatment (D/ year). **B,** Effects of atropine on axial length changes in the first and second year of treatment (mm/ year). CI= confidence interval; SD= standard deviation.

**(A)**

**
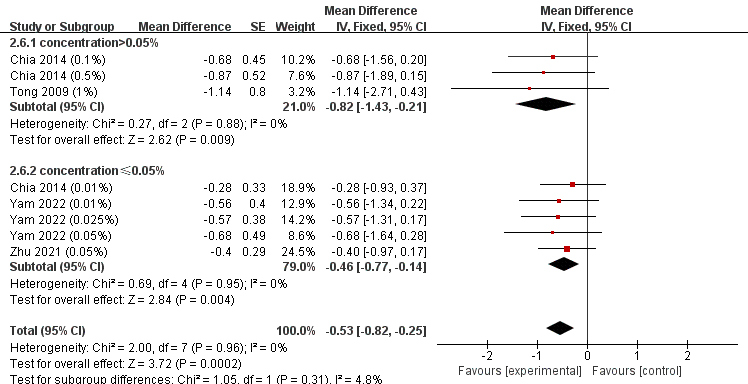
**

**(B)**

**
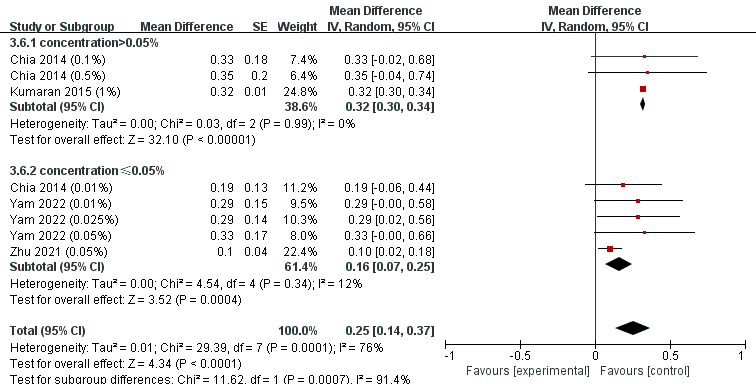
**

**Supplementary Figure 6.** **Rebound effect of myopia control effect in washout stage. A,** Rebound effect of atropine effect on refraction change in washout stage (D/ year)**. B,** Rebound effect of atropine effect on axial length change in washout stage (mm/ year)**.** CI= confidence interval; SE= standard errors.

**(A)**

**
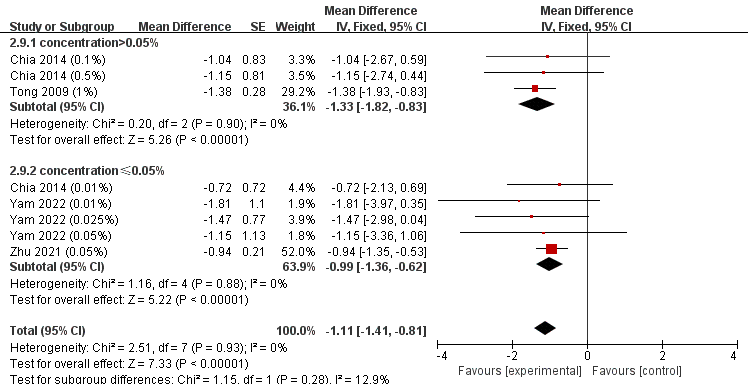
**

**(B)**

**
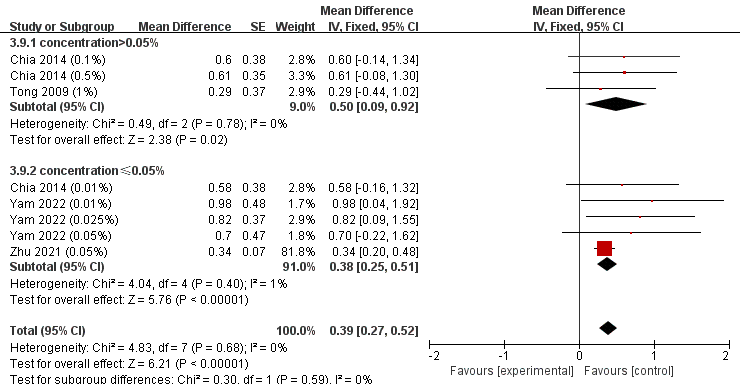
**

**Supplementary Figure 7.** **Effect of atropine on refraction change and axial length change in all 3 years. A,** Effect of atropine on refraction change in all 3 years (D). **B,** Effect of atropine on axial length change in all 3 years (mm). CI= confidence interval; SE= standard errors.

**(A)**

**
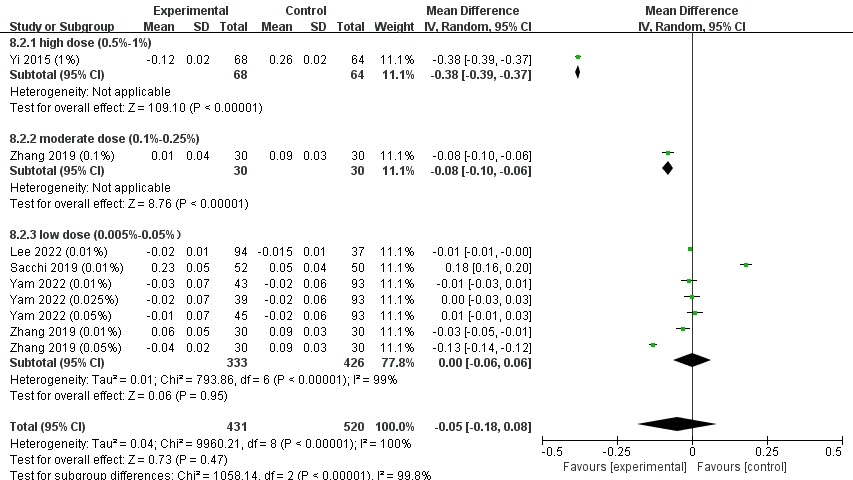
**

**(B****)**

**
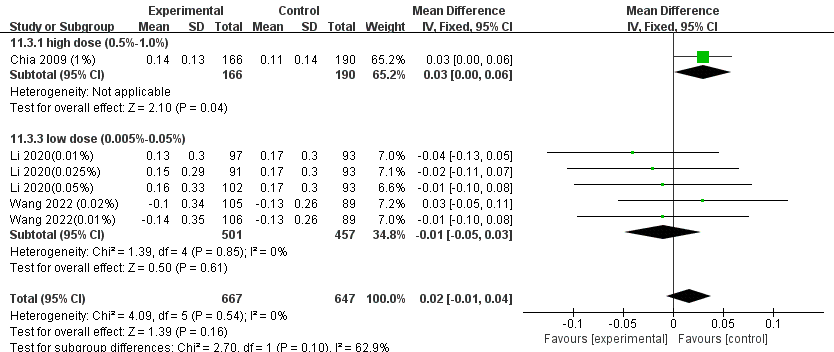
**

**(C)**

**
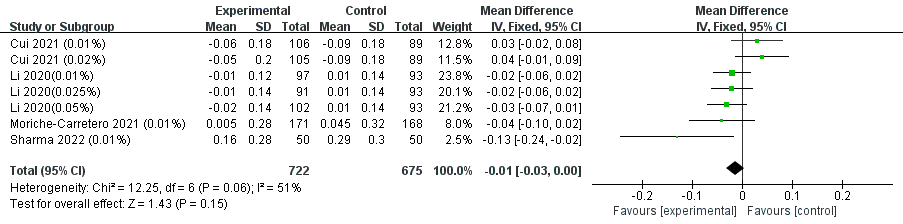
**

**(D)**

**
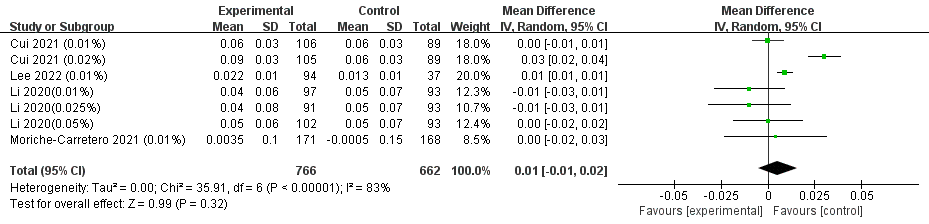
**

**(E)**

**
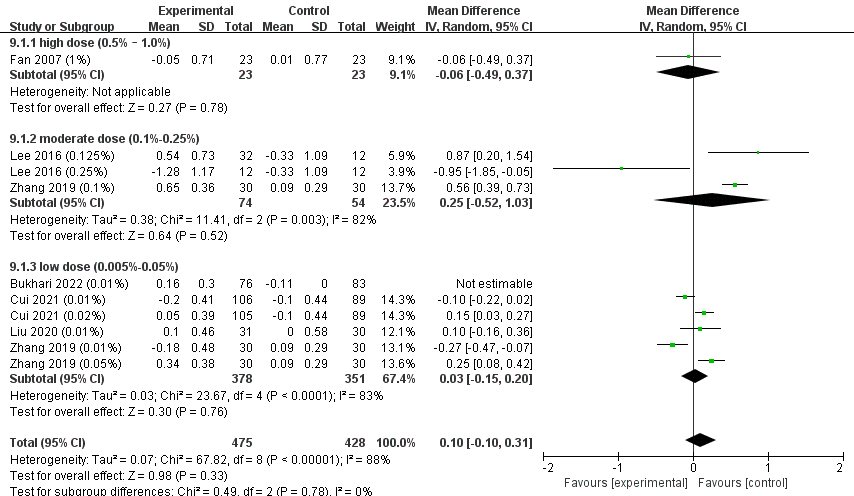
**

**Supplementary Figure 8.** **Effect of atropine on change in BCVA, astigmatism, anterior segment (corneal curvature and ACD), and IOP. A,** Effect of atropine on change in BCVA (logMAR/ year). **B,** Effect of atropine on change in corneal astigmatism (D/ year). **C,** Effect of atropine on change in corneal curvature (D/ year). **D,** Effect of atropine on change in ACD (mm/year). **E,** Effect of atropine on change in IOP (mmHg/ year). CI= confidence interval; SD= standard deviation; BCVA= best corrected visual acuity; ACD= anterior chamber depth; IOP= intraocular pressure.

**(A)**

**
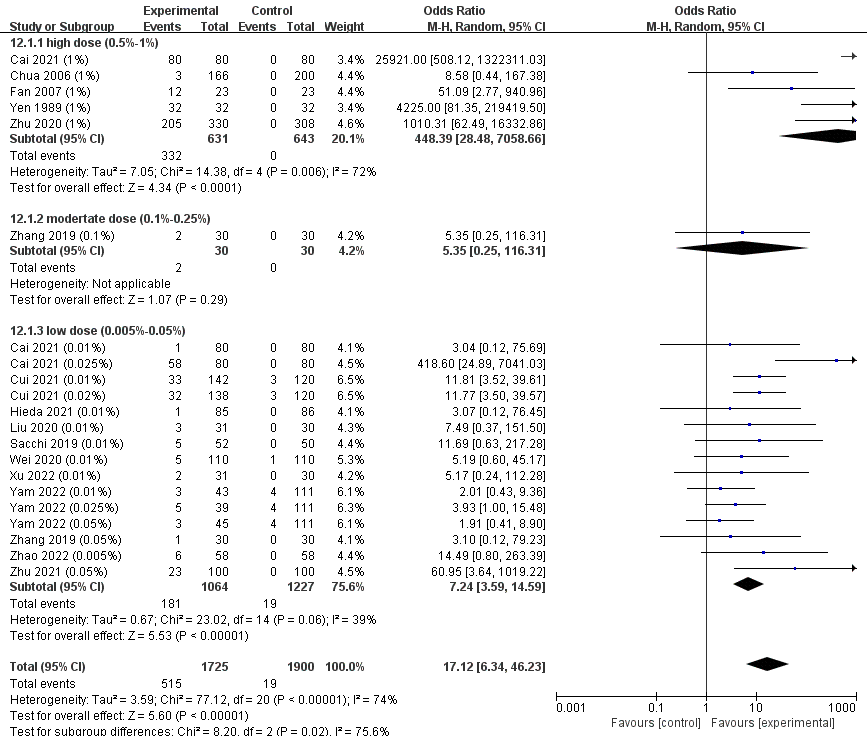
**

**(B)**

**
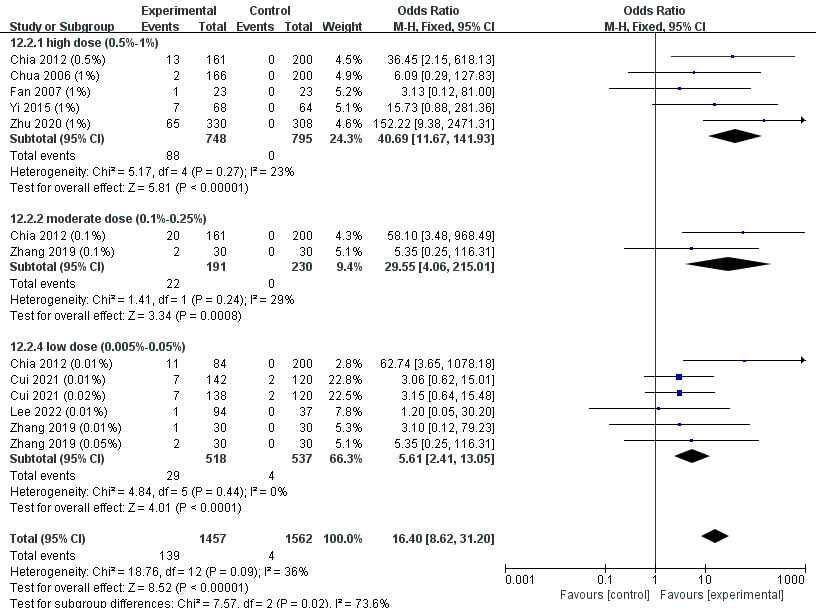
**

**(C)**

**
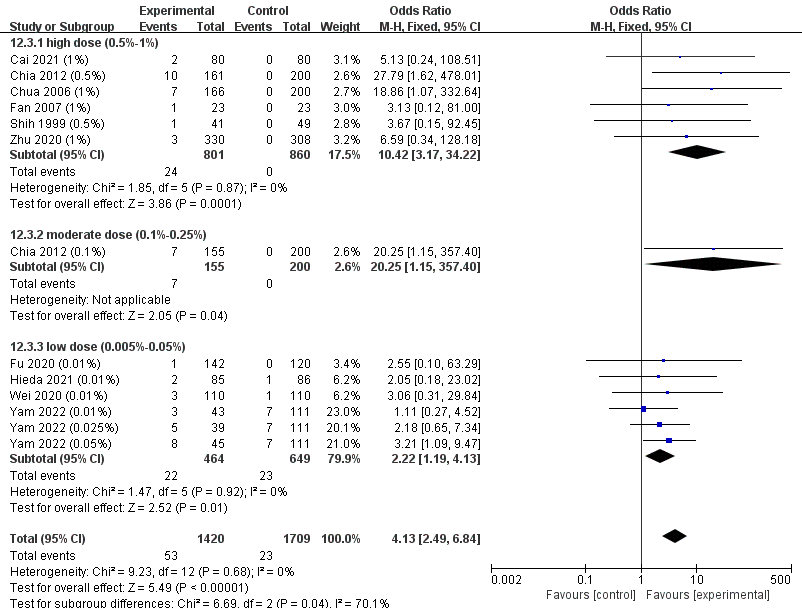
**

**Supplementary Figure 9.** **Effect of atropine on risk of photophobia, blurred near vision and allergic reactions.** **A,** Effect of atropine on risk of photophobia. **B,** Effect of atropine on risk of blurred near vision. **C,** Effect of atropine on risk of allergic reactions. CI=confidence interval.

| **(A)** | **(B)** |
| --- | --- |
| 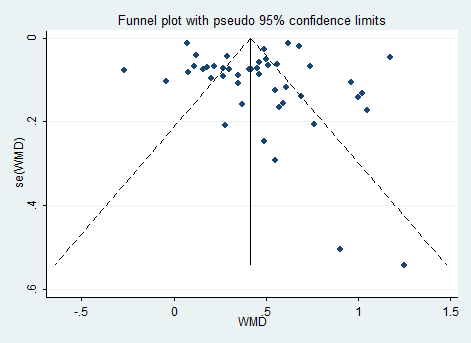 | 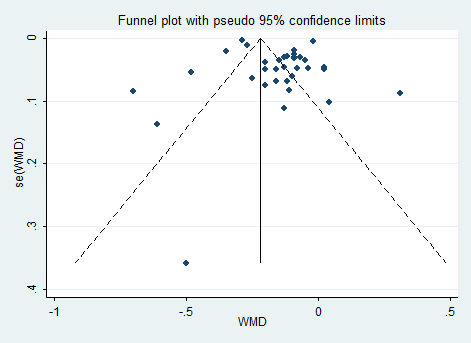 |
| **(C)** | **(D)** |
| 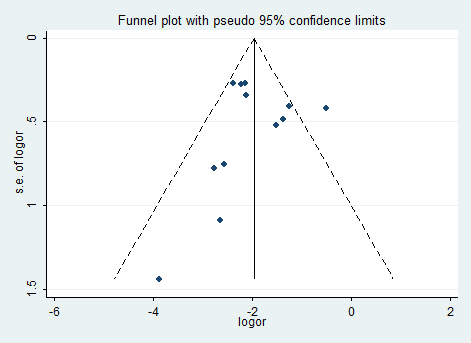 | 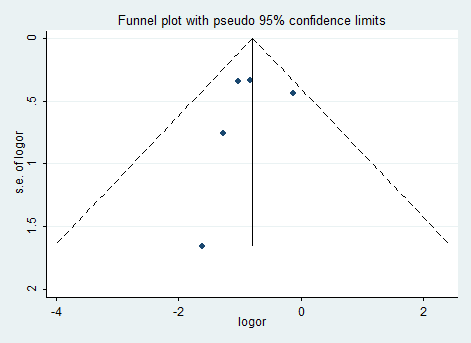 |
| **(E)** | **(F)** |
| **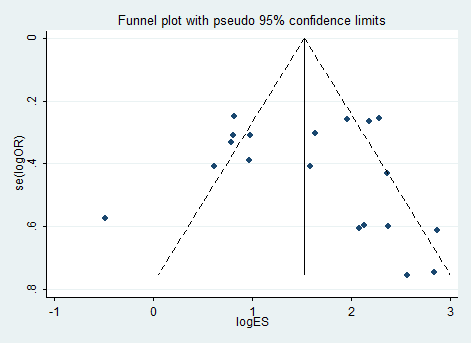** | **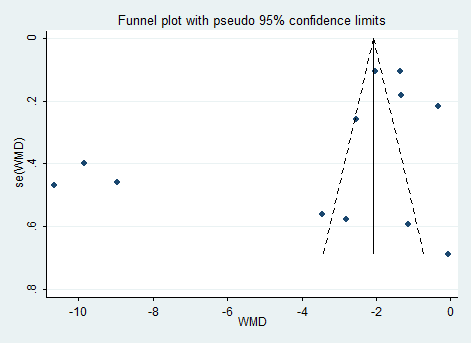** |
| **(G)** | **(H)** |
| **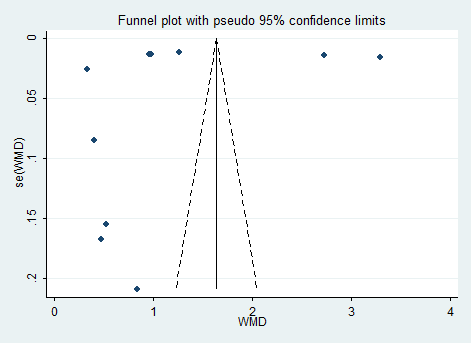** | **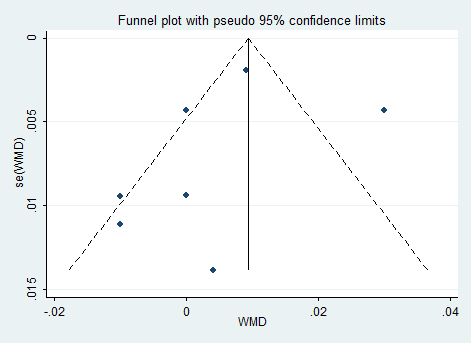** |
| **(J)** | **(K)** |
| **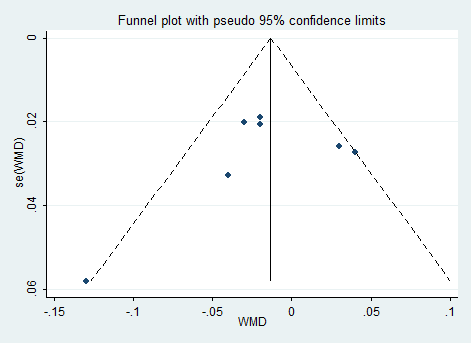** | **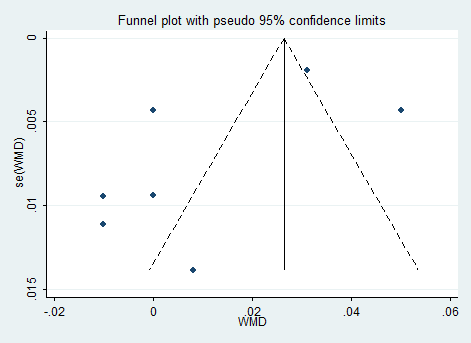** |
| **(L)** | **(M)** |
| **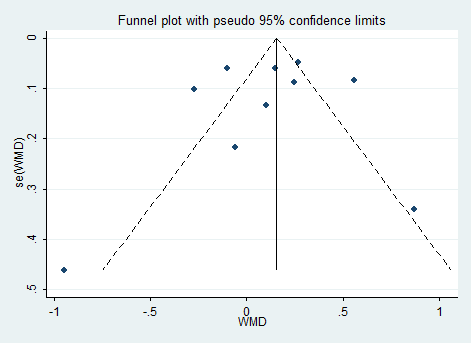** | **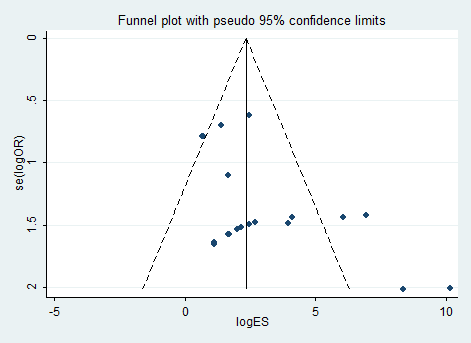** |
| **(N)** | **(O)** |
| **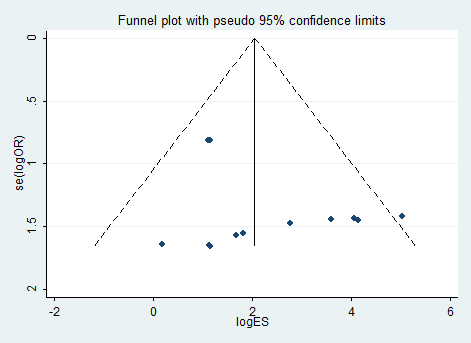** | **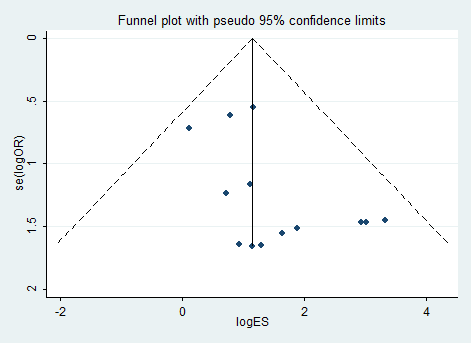** |

**Supplementary Figure 10.** **Funnel plots for bias of publication. A,** Effect of atropine on annual refraction change (D/ year). **B,** Effect of atropine on annual axial length change (mm/ year). **C,** Effect of atropine on risk of rapid myopia progression (>1.0 D/ year) (RCTs only). **D,** Effect of atropine on risk of rapid myopia progression (>1.0 D/ year) (cohort studies only). **E,** Effect of atropine on risk of slow myopia progression (<0.5 D/ year). **F,** Effect of atropine on accommodation change (D). **G,** Effect of atropine on photopic pupil diameter change (mm). **H,** Effect of atropine on change in BCVA (logMAR/ year). **I,** Effect of atropine on change in corneal astigmatism (D/ year). **J,** Effect of atropine on change in corneal curvature (D/ year). **K,** Effect of atropine on change in ACD (mm/year). **L,** Effect of atropine on change in IOP (mmHg/ year). **M,** Effect of atropine on risk of photophobia. **N,** Effect of atropine on risk of blurred near vision. **O,** Effect of atropine on risk of allergic reactions. WMD= weighted mean difference; BCVA= best corrected visual acuity; ACD= anterior chamber depth; IOP= intraocular pressure.

## Supplementary Tables

**Supplementary Table 1.** **PRISMA 2020 Checklist.**

| **Section and Topic** | **Item #** | **Checklist item** | **Location where item is reported** |  |  |
| --- | --- | --- | --- | --- | --- |
| **TITLE** | | |  |  |  |
| Title | 1 | Identify the report as a systematic review. | Page 1 |  |  |
| **ABSTRACT** | | |  |  |  |
| Abstract | 2 | See the PRISMA 2020 for Abstracts checklist. | Page 1 |  |  |
| **INTRODUCTION** | | |  |  |  |
| Rationale | 3 | Describe the rationale for the review in the context of existing knowledge. | Page 2 |  |  |
| Objectives | 4 | Provide an explicit statement of the objective(s) or question(s) the review addresses. | Page 2 |  |  |
| **METHODS** | | |  |  |  |
| Eligibility criteria | 5 | Specify the inclusion and exclusion criteria for the review and how studies were grouped for the syntheses. | Page 2-3 |  |  |
| Information sources | 6 | Specify all databases, registers, websites, organisations, reference lists and other sources searched or consulted to identify studies. Specify the date when each source was last searched or consulted. | Page 2 |  |  |
| Search strategy | 7 | Present the full search strategies for all databases, registers and websites, including any filters and limits used. | Supplementary Table 2 |  |  |
| Selection process | 8 | Specify the methods used to decide whether a study met the inclusion criteria of the review, including how many reviewers screened each record and each report retrieved, whether they worked independently, and if applicable, details of automation tools used in the process. | Page 2 |  |  |
| Data collection process | 9 | Specify the methods used to collect data from reports, including how many reviewers collected data from each report, whether they worked independently, any processes for obtaining or confirming data from study investigators, and if applicable, details of automation tools used in the process. | Page 3 |  |  |
| Data items | 10a | List and define all outcomes for which data were sought. Specify whether all results that were compatible with each outcome domain in each study were sought (e.g. for all measures, time points, analyses), and if not, the methods used to decide which results to collect. | Page 3 |  |  |
|  | 10b | List and define all other variables for which data were sought (e.g. participant and intervention characteristics, funding sources). Describe any assumptions made about any missing or unclear information. | Page 3 |  |  |
| Study risk of bias assessment | 11 | Specify the methods used to assess risk of bias in the included studies, including details of the tool(s) used, how many reviewers assessed each study and whether they worked independently, and if applicable, details of automation tools used in the process. | Page 3 |  |  |
| Effect measures | 12 | Specify for each outcome the effect measure(s) (e.g. risk ratio, mean difference) used in the synthesis or presentation of results. | Page 3 |  |  |
| Synthesis methods | 13a | Describe the processes used to decide which studies were eligible for each synthesis (e.g. tabulating the study intervention characteristics and comparing against the planned groups for each synthesis (item #5)). | Page 3 |  |  |
|  | 13b | Describe any methods required to prepare the data for presentation or synthesis, such as handling of missing summary statistics, or data conversions. | Page 3 |  |  |
|  | 13c | Describe any methods used to tabulate or visually display results of individual studies and syntheses. | Page 3 |  |  |
|  | 13d | Describe any methods used to synthesize results and provide a rationale for the choice(s). If meta-analysis was performed, describe the model(s), method(s) to identify the presence and extent of statistical heterogeneity, and software package(s) used. | Page 3 |  |  |
|  | 13e | Describe any methods used to explore possible causes of heterogeneity among study results (e.g. subgroup analysis, meta-regression). | Page 3 |  |  |
|  | 13f | Describe any sensitivity analyses conducted to assess robustness of the synthesized results. | Page 3 |  |  |
| Reporting bias assessment | 14 | Describe any methods used to assess risk of bias due to missing results in a synthesis (arising from reporting biases). | Page 3 |  |  |
| Certainty assessment | 15 | Describe any methods used to assess certainty (or confidence) in the body of evidence for an outcome. | Page 3 |  |  |
| **RESULTS** | | |  |  |  |
| Study selection | 16a | Describe the results of the search and selection process, from the number of records identified in the search to the number of studies included in the review, ideally using a flow diagram. | Page 3, Figure 1 |  |  |
|  | 16b | Cite studies that might appear to meet the inclusion criteria, but which were excluded, and explain why they were excluded. | Supplementary Table 3 |  |  |
| Study characteristics | 17 | Cite each included study and present its characteristics. | Supplementary Table 4 |  |  |
| Risk of bias in studies | 18 | Present assessments of risk of bias for each included study. | Supplementary Table 5 |  |  |
| Results of individual studies | 19 | For all outcomes, present, for each study: (a) summary statistics for each group (where appropriate) and (b) an effect estimate and its precision (e.g. confidence/credible interval), ideally using structured tables or plots. | Figure 2-6, Supplementary Figure 1-9 |  |  |
| 7Results of syntheses | 20a | For each synthesis, briefly summarise the characteristics and risk of bias among contributing studies. | Page 3 |  |  |
|  | 20b | Present results of all statistical syntheses conducted. If meta-analysis was done, present for each the summary estimate and its precision (e.g. confidence/credible interval) and measures of statistical heterogeneity. If comparing groups, describe the direction of the effect. | Page 3-10 |  |  |
|  | 20c | Present results of all investigations of possible causes of heterogeneity among study results. | Page 3-10 |  |  |
|  | 20d | Present results of all sensitivity analyses conducted to assess the robustness of the synthesized results. | Page 10 |  |  |
| Reporting biases | 21 | Present assessments of risk of bias due to missing results (arising from reporting biases) for each synthesis assessed. | Page 10 |  |  |
| Certainty of evidence | 22 | Present assessments of certainty (or confidence) in the body of evidence for each outcome assessed. | Page 10 |  |  |
| **DISCUSSION** | | |  |  |  |
| Discussion | 23a | Provide a general interpretation of the results in the context of other evidence. | Page 10-11 |  |  |
|  | 23b | Discuss any limitations of the evidence included in the review. | Page 11 |  |  |
|  | 23c | Discuss any limitations of the review processes used. | Page 11 |  |  |
|  | 23d | Discuss implications of the results for practice, policy, and future research. | Page 11-12 |  |  |
| **OTHER INFORMATION** | | |  |  |  |
| Registration and protocol | 24a | Provide registration information for the review, including register name and registration number, or state that the review was not registered. | Page 2 |  |  |
|  | 24b | Indicate where the review protocol can be accessed, or state that a protocol was not prepared. | NA |  |  |
|  | 24c | Describe and explain any amendments to information provided at registration or in the protocol. | NA |  |  |
| Support | 25 | Describe sources of financial or non-financial support for the review, and the role of the funders or sponsors in the review. | Page 12 |  |  |
| Competing interests | 26 | Declare any competing interests of review authors. | Page 12 |  |  |
| Availability of data, code and other materials | 27 | Report which of the following are publicly available and where they can be found: template data collection forms; data extracted from included studies; data used for all analyses; analytic code; any other materials used in the review. | NA |  |  |

*****NA=Not Available.

*From:*  Page MJ, McKenzie JE, Bossuyt PM, Boutron I, Hoffmann TC, Mulrow CD, et al. The PRISMA 2020 statement: an updated guideline for reporting systematic reviews. BMJ 2021;372:n71. doi: 10.1136/bmj.n71

**Supplementary Table 2.** **Search Strategy.**

(1) Pubmed

| Search order | Query |
| --- | --- |
| #1  #2  #3  #4 #5 #6  #7  #8 #9 | Myopia[Mesh]  Refractive errors[Mesh]  Myopia[Mesh] OR Myopia[Tiab] OR Myopic[Tiab] OR Refractive errors[Mesh] OR “Refractive errors” [Tiab] OR “Refractive error” [Tiab] OR ((short OR near*) AND sight*) (short OR near*) AND sight*  Mydriatics[MeSH]  “Muscarinic Antagonists”[MeSH]  “Cholinergic Antagonists”[MeSH]  Mydriatics[MeSH] OR mydriat* OR “Muscarinic Antagonists”[MeSH] OR (muscarinic AND antagonist*) OR (anti AND muscarinic) OR “Cholinergic Antagonists”[MeSH] OR (cholinergic AND antagonist*) OR (anti AND cholinergic) OR atropine*)  progress* or slow* or retard* or effect* or treat* or efficacy*  #3 AND #7 AND #8 |

(2) Cochrane library

| Search order | Query |
| --- | --- |
| #1  #2  #3  #4 #5 #6  #7  #8 #9 #10 #11 #12 #13  #14  #15  #16 #17 #18 | MeSH descriptor myopia (short OR near*) next sight* myop* MeSH descriptor Refractive Errors refract* (#1 OR #2 OR #3 OR #4 OR #5) MeSH descriptor Mydriatics mydriat* MeSH descriptor Muscarinic Antagonists muscarinic next antagonist* anti next muscarinic MeSH descriptor Cholinergic Antagonists cholinergic next antagonist* anti next cholinergic atropine*1  (#7 OR #8 OR #9 OR #10 OR #11 OR #12 OR #13 OR #14 OR #15) progress* OR slow* OR retard* OR effect* OR treat* OR efficacy* (#6 AND #16 AND #17) |

(3) Embase

| Search order | Query |
| --- | --- |
| #1  #2  #3  #4 #5  #6  #7  #8 #9 | ‘Myopia’/exp ‘Myopic’  ‘Refraction error’/exp OR ‘Refractive error’ OR ‘Refractive errors’  ‘shortsighted’/exp  #1 OR #2 OR #3 OR #4  ‘mydriatic agent’/exp OR ‘mydriat*’ OR ‘Muscarinic Receptor Blocking Agent’/exp OR ‘muscarinic antagonist’ OR ‘Cholinergic Receptor Blocking Agent’/exp OR ‘anti cholinergic’ OR ‘Atropine’  progress* OR slow* OR retard* OR effect* OR treat* OR efficacy*  #6 AND #7  #5 AND #8 |

**Supplementary Table 3.** **Exclusion studies list.**

| Study | Reasons for exclusion |
| --- | --- |
| Lee 2006(Lee et al., 2006) | The duration of medication was uncertain, with the lowest being 9 months and no more than a year. And the follow-up duration of the experimental group and the control group is not completely the same. |
| Chiang 2001(Chiang et al., 2001) | No control group. |
| Chou 1997(Chou et al., 1997) | The refractive progression status of the treatment group before atropine treatment was used as the control. |
| Cooper 2013(Cooper et al., 2013) | No control group. |
| Luu 2005(Luu et al., 2005) | Only reported electroretinogram data. |
| Fang 2010(Fang et al., 2010) | Population included in the study is premyopes. |
| Wu 2011(Wu et al., 2011) | The dose of atropine was adjusted halfway of the study individually according to the myopic progression rate of the patients. |
| Brodstein 1984(Brodstein et al., 1984) | The data presented in the article are shown stratified by age and cannot be pooled. |
| Wu 2012(Wu et al., 2012) | Corss-sectional study. |
| Chia 2013(Chia et al., 2013) | It is a subset of the ATOM2 and mainly reported electroretinogram data. |
| Erdinest 2022(Erdinest et al., 2021a) | No placebo or blank control group: evaluating the effect of COVID-19 pandemic on 0.01% atropine treatment for myopia control |
| Erdinest 2021(Erdinest et al., 2021b) | A case series. |
| Clark 2015(Clark and Clark, 2015) | Follow-up duration is not unified, and the minimum follow-up time is too short, no more than one year. |
| Chan 2017(Chan et al., 2017) | No control group. |
| Cheng 2020(Cheng et al., 2020) | No control group and the follow-up duration is too short, only a half year. Evaluating the effect of 0.01% atropine eye drops on the ocular surface in children for the control of myopia |
| Chuang 2021(Chuang et al., 2021) | No placebo or blank control group and the dosage of atropine was changed according to the myopic progression of each patient. |
| Foo 2020(Foo et al., 2020) | No placebo or blank control group: comparing different part-time use of 1% atropine eye drops regimen on myopic progression. |
| Fu 2021(Fu et al., 2021) | No placebo or blank control group and no myopic progression rate reported |
| Fu 2018(Fu et al., 2018) | The follow-up time is only 4 months, no more than one year. |
| Ho 2020(Ho et al., 2020) | No placebo or blank control group and the follow-up duration is too short, only a week. |
| Jeon 2021(Jeon et al., 2021) | No placebo or blank control group: comparing the parameters of the good responders and poor responder. |
| Ji 2022(Ji et al., 2022) | No placebo control group: comparing the effect of orthokeratology and atropine. |
| Jiang 2021(Jiang et al., 2021) | The follow-up time is only a week, no more than one year. |
| Jones 2022(Jones et al., 2022) | No placebo or blank control group: comparing the effect of soft multifocal contact lenses and its combination with atropine. |
| Kao 2021(Kao et al., 2021) | The follow-up time is too short, no more than one year. |
| Kong 2021(Kong et al., 2021) | No placebo or blank control group: comparing the effect of auricular acupoints and its combination with atropine. |
| Kothari 2017(Kothari and Rathod, 2017) | No control group. |
| Lee 2022(Lee et al., 2022) | No control group. |
| Li 2022(Li et al., 2022a) | No control group. |
| Li 2021(Li et al., 2021b) | No placebo or blank control group: comparing the effect of orthokeratology and its combination with atropine. |
| Li 2022(Li et al., 2022b) | No placebo or blank control group: comparing the effect of different dosage of atropine regimen on myopic control. |
| Li 2020(Li et al., 2020) | The follow-up time is too short, no more than one year. |
| Loh 2015(Loh and Chia, 2015) | Duplicate data. |
| Lyu 2021(Lyu et al., 2021) | No placebo or blank control group: comparing the effect of orthokeratology and atropine. |
| Moon 2018(Moon and Shin, 2018) | No placebo or blank control group: comparing the effect of different dosage of atropine regimen on myopic control. |
| Myles 2021(Myles et al., 2021) | No placebo or blank control group. |
| Nishiyama 2015(Nishiyama et al., 2015) | No placebo or blank control group. |
| Pérez-Flores 2021(Pérez-Flores et al., 2021) | No placebo or blank control group. |
| Polling 2016(Polling et al., 2016) | No placebo or blank control group. |
| Polling 2020(Polling et al., 2020^)^ | No placebo or blank control group. |
| Qin 2021(Qin et al., 2021) | No placebo or blank control group: comparing the effect of different dosage of atropine regimen on myopic control. |
| Rose 2021(Rose et al., 2021) | No placebo or blank control group. |
| Tang 2020(Tang et al., 2020) | No placebo or blank control group: investigating the effect of combination of orthokeratology and atropine on myopic control. |
| Tsai 2020(Tsai et al., 2020) | No placebo or blank control group: comparing the effect of orthokeratology and atropine on myopic progression. |
| Verzhanskaya 2017(Verzhanskaya and Tarutta, 2017) | No placebo or blank control group. |
| Vincent 2020(Vincent et al., 2020) | The follow-up time is too short, no more than one year. |
| Wang 2017(Wang, 2017) | No placebo or blank control group: comparing the effect of raceanisodamine and atropine on myopic progression. |
| Wang 2022(Wang et al., 2022) | The follow-up time is too short, no more than one year. |
| Zhang 2020(Zhang et al., 2020) | No placebo or blank control group. |
| Zhou 2022(Zhou et al., 2022) | No placebo or blank control group: comparing the effect of orthokeratology and its combination with atropine. |
| Lin 2021(Lin et al., 2021) | Duplicate data. |
| Cheng 2021(Cheng et al., 2021) | The follow-up time is too short, no more than one year. |
| Jiang 2022(Jiang et al., 2022) | The follow-up time is only 3 months, no more than one year. |
| Li 2021(Li et al., 2021a) | Exploring the effect of age on myopia control with different concentrations of atropine |
| Zhao 2021(Zhao et al., 2021) | The follow-up time is too short, no more than one year. |
| Zhao 2021^(^Zhao and Hao, 2021) | No placebo control group: comparing the effect of orthokeratology and atropine |
| Ye 2020(Ye et al., 2020) | The follow-up time is only a half year, no more than one year. |
| Ye 2021(Ye et al., 2021) | No placebo control group: comparing the effect of cyclopentolate and atropine |
| Jethani 2022(Jethani, 2022) | Population included in the study is premyopes. |
| Wan 2018(Wan et al., 2018) | No placebo control group: comparing the effect of orthokeratology and atropine. |
| Luo 2021(Luo et al., 2021) | No placebo control group: comparing the effect of orthokeratology and atropine. |
| Tan 2020(Tan et al., 2020) | No placebo or blank control group: comparing the effect of orthokeratology and its combination with atropine. |
| Shih 2001(Shih et al., 2001) | No specific inclusion criteria for refraction were reported. |
| Wang 2017(Wang et al., 2017) | It is not clear whether refraction measurements was under cycloplegia during follow-up duration. |
| Han 2019(Han et al., 2019) | The refraction range mentioned in the inclusion criteria was beyond the inclusion range in our study. |
| Meng 2020(Meng, 2020) | Part of the included patients were premyopes. |
| Rong 2020(Rong et al., 2020) | Duplicate data of Fu 2020. |
| Zhao 2015(Zhao et al., 2015) | No specific inclusion criteria for refraction were reported. |
| Erdinest 2022(Erdinest et al., 2022) | NOS score is lower than 7. |
| Wang 2023(Wang et al., 2023) | Duplicate data of Cui 2020. |
| Larkin 2019(Larkin et al., 2019) | The refraction range mentioned in the inclusion criteria was beyond the inclusion range in our study. |
| Saxena 2021(Saxena et al., 2021) | It is not clear whether refraction measurements were under cycloplegia during follow-up duration. |
| Ren 2017(Ren et al., 2017) | No specific inclusion criteria for refraction were reported. |
| Wang 2021(Wang et al., 2021) | No specific inclusion criteria for refraction were reported. |
| Shen 2020(Shen and Meng, 2020) | It is not clear whether refraction measurements were under cycloplegia during follow-up duration. |
| Zheng 2022(Zheng et al., 2022) | It is not clear whether refraction measurements were under cycloplegia and no description of duration of follow-ups. |

**Reference:**

Brodstein, R.S., Brodstein, D.E., and Olson, R.J. (1984). The treatment of myopia with atropine and bifocals. A long-term prospective study. *Ophthalmology* 91(11)**,** 1373-1379.

Chan, L.W., Hsieh, Y.T., Hsu, W.C., Cheng, H.C., and Shen, E.P. (2017). Optic Disc Parameters of Myopic Children with Atropine Treatment. *Curr. Eye Res.* 42(12)**,** 1614-1619. doi: 10.1080/02713683.2017.1359846.

Cheng, J., Yang, Y., Kong, X., Zeng, L., Chen, Z., Xu, J., et al. (2020). The Effect of 0.01% Atropine Eye Drops on the Ocular Surface in Children for the Control of Myopia-The Primary Results from a Six-Month Prospective Study. *Ther. Clin. Risk Manag.* 16**,** 735-740. doi: 10.2147/tcrm.S265945.

Cheng, Z., Mei, J., Cao, S., Zhang, R., Zhou, J., and Wang, Y. (2021). The Effects of 0.01% Atropine on Adult Myopes' Contrast Sensitivity. *Front. Neurosci.* 15**,** 624472. doi: 10.3389/fnins.2021.624472.

Chia, A., Li, W., Tan, D., and Luu, C.D. (2013). Full-field electroretinogram findings in children in the atropine treatment for myopia (ATOM2) study. *Doc. Ophthalmol.* 126(3)**,** 177-186. doi: 10.1007/s10633-012-9372-8.

Chiang, M.F., Kouzis, A., Pointer, R.W., and Repka, M.X. (2001). Treatment of childhood myopia with atropine eyedrops and bifocal spectacles. *Binocul. Vis. Strabismus Q.* 16(3)**,** 209-216.

Chou, A.C., Shih, Y.F., Ho, T.C., and Lin, L.L. (1997). The effectiveness of 0.5% atropine in controlling high myopia in children. *J. Ocul. Pharmacol. Ther.* 13(1)**,** 61-67. doi: 10.1089/jop.1997.13.61.

Chuang, M.N., Fang, P.C., and Wu, P.C. (2021). Stepwise low concentration atropine for myopic control: a 10-year cohort study. *Sci. Rep.* 11(1)**,** 17344. doi: 10.1038/s41598-021-96698-6.

Clark, T.Y., and Clark, R.A. (2015). Atropine 0.01% Eyedrops Significantly Reduce the Progression of Childhood Myopia. *J. Ocul. Pharmacol. Ther.* 31(9)**,** 541-545. doi: 10.1089/jop.2015.0043.

Cooper, J., Eisenberg, N., Schulman, E., and Wang, F.M. (2013). Maximum atropine dose without clinical signs or symptoms. *Optom. Vis. Sci.* 90(12)**,** 1467-1472.

Erdinest, N., London, N., Lavy, I., Landau, D., Ben Ephraim Noyman, D., Levinger, N., et al. (2022). Low-Concentration Atropine Monotherapy vs. Combined with MiSight 1 Day Contact Lenses for Myopia Management. *Vision (Basel)* 6(4). doi: 10.3390/vision6040073.

Erdinest, N., London, N., Levinger, N., Lavy, I., Pras, E., and Morad, Y. (2021a). Decreased effectiveness of 0.01% atropine treatment for myopia control during prolonged COVID-19 lockdowns. *Contact Lens and Anterior Eye*. doi: 10.1016/j.clae.2021.101475.

Erdinest, N., London, N., Levinger, N., and Morad, Y. (2021b). Myopia Control with Combination Low-Dose Atropine and Peripheral Defocus Soft Contact Lenses: A Case Series. *Case Report. Ophthalmol.* 12(2)**,** 548-554. doi: 10.1159/000515568.

Fang, P.C., Chung, M.Y., Yu, H.J., and Wu, P.C. (2010). Prevention of myopia onset with 0.025% atropine in premyopic children. *J. Ocul. Pharmacol. Ther.* 26(4)**,** 341-345. doi: 10.1089/jop.2009.0135.

Foo, L.L., Htoon, H., Farooqui, S.Z., and Chia, A. (2020). Part-time use of 1% atropine eye drops for prevention of myopia progression in children. *Int. Ophthalmol.* 40(7)**,** 1857-1862. doi: 10.1007/s10792-020-01356-x.

Fu, A., Stapleton, F., Wei, L., Wang, W., Zhao, B., Watt, K., et al. (2021). Risk factors for rapid axial length elongation with low concentration atropine for myopia control. *Sci. Rep.* 11(1)**,** 11729. doi: 10.1038/s41598-021-88719-1.

Fu, A., Zhao, B., Zhang, J., Zhang, J., Li, B., Li, X., et al. (2018). Adverse effects of 0.01% atropine sulfate eye drops on myopic children. *Zhonghua Shiyan Yanke Zazhi/Chinese Journal of Experimental Ophthalmology* 36(6)**,** 449-453. doi: 10.3760/cma.j.issn.2095-0160.2018.06.010.

Han, W.T., Rong, A., and Xu, W. (2019). [Combination with different anticholinergic eyedrops for the treatment of children myopia]. *Zhonghua Yi Xue Za Zhi* 99(24)**,** 1859-1863. doi: 10.3760/cma.j.issn.0376-2491.2019.24.005.

Ho, M.C., Hsieh, Y.T., Shen, E.P., Hsu, W.C., and Cheng, H.C. (2020). Short-term refractive and ocular parameter changes after topical atropine. *Taiwan J Ophthalmol* 10(2)**,** 111-115. doi: 10.4103/tjo.tjo_110_18.

Jeon, G.S., Hong, I.H., Lee, J.H., Song, T.G., Lee, T.Y., and Han, J.R. (2021). Analysis of treatment response about low-dose (0.01%) atropine eye-drops in myopic children. *Eur. J. Ophthalmol.* doi: 10.1177/11206721211038817.

Jethani, J. (2022). Efficacy of low-concentration atropine (0.01%) eye drops for prevention of axial myopic progression in premyopes. *Indian J. Ophthalmol.* 70(1)**,** 238-240. doi: 10.4103/ijo.IJO_1462_21.

Ji, N., Niu, Y., Qin, J., Fu, A.C., and Cui, C. (2022). Orthokeratology Lenses Versus Administration of 0.01% Atropine Eye Drops for Axial Length Elongation in Children With Myopic Anisometropia. *Eye & contact lens* 48(1)**,** 45-50. doi: 10.1097/ICL.0000000000000848.

Jiang, J., Long, W., Hu, Y., Zhao, F., Zhao, W., Zheng, B., et al. (2022). Accommodation and vergence function in children using atropine combined with orthokeratology. *Cont Lens Anterior Eye***,** 101704. doi: 10.1016/j.clae.2022.101704.

Jiang, Y., Zhang, Z., Wu, Z., Sun, S., Fu, Y., and Ke, B. (2021). Change and Recovery of Choroid Thickness after Short-term Application of 1% Atropine Gel and Its Influencing Factors in 6-7-year-old Children. *Curr. Eye Res.* 46(8)**,** 1171-1177. doi: 10.1080/02713683.2020.1863431.

Jones, J.H., Mutti, D.O., Jones-Jordan, L.A., and Walline, J.J. (2022). Effect of Combining 0.01% Atropine with Soft Multifocal Contact Lenses on Myopia Progression in Children. *Optom. Vis. Sci.* 99(5)**,** 434-442. doi: 10.1097/OPX.0000000000001884.

Kao, P.H., Chuang, L.H., Lai, C.C., Chen, S.Y., Lin, K.K., Lee, J.S., et al. (2021). Evaluation of axial length to identify the effects of monocular 0.125% atropine treatment for pediatric anisometropia. *Sci. Rep.* 11(1)**,** 21511. doi: 10.1038/s41598-021-96414-4.

Kong, X.H., Zhao, Y., Chen, Z., Zeng, L., Han, R., Dong, X.Q., et al. (2021). A Randomized Controlled Trial of the Effect of 0.01% Atropine Eye Drops Combined with Auricular Acupoint Stimulation on Myopia Progression. *J. Ophthalmol.* 2021**,** 5585441. doi: 10.1155/2021/5585441.

Kothari, M., and Rathod, V. (2017). Efficacy of 1% atropine eye drops in retarding progressive axial myopia in Indian eyes. *Indian J. Ophthalmol.* 65(11)**,** 1178-1181. doi: 10.4103/ijo.IJO_418_17.

Larkin, G.L., Tahir, A., Epley, K.D., Beauchamp, C.L., Tong, J.T., and Clark, R.A. (2019). Atropine 0.01% Eye Drops for Myopia Control in American Children: A Multiethnic Sample Across Three US Sites. *Ophthalmol. Ther.* 8(4)**,** 589-598. doi: 10.1007/s40123-019-00217-w.

Lee, J.J., Fang, P.C., Yang, I.H., Chen, C.H., Lin, P.W., Lin, S.A., et al. (2006). Prevention of myopia progression with 0.05% atropine solution. *J. Ocul. Pharmacol. Ther.* 22(1)**,** 41-46. doi: 10.1089/jop.2006.22.41.

Lee, L.C., Hsieh, M.W., Chen, Y.H., Chen, P.L., and Chien, K.H. (2022). Characteristics of responders to atropine 0.01% as treatment in Asian myopic children. *Sci. Rep.* 12(1)**,** 7380. doi: 10.1038/s41598-022-10978-3.

Li, F.F., Zhang, Y., Zhang, X., Yip, B.H.K., Tang, S.M., Kam, K.W., et al. (2021a). Age Effect on Treatment Responses to 0.05%, 0.025%, and 0.01% Atropine: Low-Concentration Atropine for Myopia Progression Study. *Ophthalmology* 128(8)**,** 1180-1187. doi: 10.1016/j.ophtha.2020.12.036.

Li, H., Zhang, L., Tian, H., Zhang, S., Zhang, X., Zhang, H., et al. (2022a). Effect of 0.01% Atropine on Accommodation in Myopic Teenagers. *Front. Pharmacol.* 13. doi: 10.3389/fphar.2022.808440.

Li, P., Liu, T., and Sun, X.T. (2021b). Effect of orthokeratology combined with 0.01% Atropine Sulfate Ophthalmic Gel on curative effect and analysis influencing factors on children's myopia. *International Eye Science* 21(4)**,** 698-701. doi: 10.3980/j.issn.1672-5123.2021.4.27.

Li, W., Cao, Y., and Zhou, J. (2022b). Effects of low-concentration atropine eye drops on the optical quality of the eyes in myopic children. *Indian J. Ophthalmol.* 70(6)**,** 2107-2110. doi: 10.4103/ijo.IJO_2886_21.

Li, W., Jiang, R., Zhu, Y., Zhou, J., and Cui, C. (2020). Effect of 0.01% atropine eye drops on choroidal thickness in myopic children. *J. Fr. Ophtalmol.* doi: 10.1016/j.jfo.2020.04.023.

Lin, X., Chen, Q., Duan, W., Zhu, Q., and Hu, M. (2021). Clinical study of intermittent use of 1% atropine on retardation of myopia progression in Chinese school children. *Chin J Sch Health* 42(2). doi: 10.16835/ j.cnki.1000-9817.2021.02.005.

Loh, K.L.L.Q.T.D., and Chia, A. (2015). Risk factors for progressive myopia in the atropine therapy for myopia study. *Am. J. Ophthalmol.* 159(5)**,** 945‐949.

Luo, Y., Luo, W.Q., Lu, P.F., Mao, H.J., Wei, D.F., and Huang, Y.Y. (2021). Clinical observation of orthokeratology combined with 0.01% Atropine for controlling low to moderate myopia in adolescents. *International Eye Science* 21(1)**,** 47-52. doi: 10.3980/j.issn.1672-5123.2021.1.09.

Luu, C.D., Lau, A.M., Koh, A.H., and Tan, D. (2005). Multifocal electroretinogram in children on atropine treatment for myopia. *Br. J. Ophthalmol.* 89(2)**,** 151-153. doi: 10.1136/bjo.2004.045526.

Lyu, Y., Ji, N., Fu, A.C., Wang, W.Q., Wei, L., Qin, J., et al. (2021). Comparison of Administration of 0.02% Atropine and Orthokeratology for Myopia Control. *Eye & contact lens* 47(2)**,** 81-85. doi: 10.1097/ICL.0000000000000699.

Meng, R. (2020). Analysis of the Effectiveness of 0.01% Atropine in Controlling Myopia Progression. *China & Foreign Medical Treatment* 39(01)**,** 96-98. doi: 10.16662/j.cnki.1674-0742.2020.01.096.

Moon, J.S., and Shin, S.Y. (2018). The diluted atropine for inhibition of myopia progression in Korean children. *Int J Ophthalmol* 11(10)**,** 1657-1662. doi: 10.18240/ijo.2018.10.13.

Myles, W., Dunlop, C., and McFadden, S.A. (2021). The effect of long-term low-dose atropine on refractive progression in myopic Australian school children. *Journal of Clinical Medicine* 10(7). doi: 10.3390/jcm10071444.

Nishiyama, Y., Moriyama, M., Fukamachi, M., Uchida, A., Miyaushiro, H., Kurata, A., et al. (2015). Side Effects of Low Dose Atropine. *Nippon Ganka Gakkai zasshi* 119(11)**,** 812-816.

Pérez-Flores, I., Macías-Murelaga, B., and Barrio-Barrio, J. (2021). A multicenter Spanish study of atropine 0.01% in childhood myopia progression. *Sci. Rep.* 11(1)**,** 21748. doi: 10.1038/s41598-021-00923-1.

Polling, J.R., Kok, R.G., Tideman, J.W., Meskat, B., and Klaver, C.C. (2016). Effectiveness study of atropine for progressive myopia in Europeans. *Eye (Lond.)* 30(7)**,** 998-1004. doi: 10.1038/eye.2016.78.

Polling, J.R., Tan, E., Driessen, S., Loudon, S.E., Wong, H.L., van der Schans, A., et al. (2020). A 3-year follow-up study of atropine treatment for progressive myopia in Europeans. *Eye (Basingstoke)* 34(11)**,** 2020-2028. doi: 10.1038/s41433-020-1122-7.

Qin, J., Lyu, Y., Wei, L., Zhang, J., Zhao, B., and Fu, A. (2021). Comparison of myopia progression between different concentrations and application frequencies of atropine eye drops in children. *Zhonghua Shiyan Yanke Zazhi/Chinese Journal of Experimental Ophthalmology* 39(5)**,** 423-429. doi: 10.3760/cma.j.cn115989-20200101-00015.

Ren, Q.J., Yue, H., Wang, P., Liu, R.J., and Lü, P. (2017). Effects of low concentration atropine and orthokeratology on myopia prevention and control. *International Eye Science* 17(4)**,** 794-796. doi: 10.3980/j.issn.1672-5123.2017.4.57.

Rong, J., Fu, A., Lyu, Y., Zhao, B., Wang, W., and Zhang, J. (2020). Clinical effect of 0.01% atropine eye drops on myopia progression in school-age children. *Zhonghua Shiyan Yanke Zazhi/Chinese Journal of Experimental Ophthalmology* 38(6)**,** 494-498. doi: 10.3760/cma.j.cn115989-20200329-00223.

Rose, L.V.T., Schulz, A.M., and Graham, S.L. (2021). Use baseline axial length measurements in myopic patients to predict the control of myopia with and without atropine 0.01. *PLoS One* 16(7)**,** e0254061. doi: 10.1371/journal.pone.0254061.

Saxena, R., Dhiman, R., Gupta, V., Kumar, P., Matalia, J., Roy, L., et al. (2021). Atropine for the Treatment of Childhood Myopia in India: Multicentric Randomized Trial. *Ophthalmology* 128(9)**,** 1367-1369. doi: 10.1016/j.ophtha.2021.01.026.

Shen, Y., and Meng, Z. (2020). Observation of the effect of 0.01% atropine in the control of myopia in children. *Shanxi Med J* 49(09)**,** 1139-1141. doi: 10.3969/j.issn.0253-9926.2020.09.030.

Shih, Y.F., Hsiao, C.K., Chen, C.J., Chang, C.W., Hung, P.T., and Lin, L.L. (2001). An intervention trial on efficacy of atropine and multi-focal glasses in controlling myopic progression. *Acta Ophthalmol. Scand.* 79(3)**,** 233-236. doi: 10.1034/j.1600-0420.2001.790304.x.

Tan, Q., Ng, A.L., Choy, B.N., Cheng, G.P., Woo, V.C., and Cho, P. (2020). One-year results of 0.01% atropine with orthokeratology (AOK) study: a randomised clinical trial. *Ophthalmic Physiol. Opt.* 40(5)**,** 557-566. doi: 10.1111/opo.12722.

Tang, W.T., Tian, M., Li, S.B., and Yu, Q. (2020). Clinical observation of low-dose Atropine combined with orthokeratology in the treatment of myopia. *International Eye Science* 20(6)**,** 1044-1047. doi: 10.3980/j.issn.1672-5123.2020.6.26.

Tsai, W.S., Wang, J.H., and Chiu, C.J. (2020). A comparative study of orthokeratology and low-dose atropine for the treatment of anisomyopia in children. *Sci. Rep.* 10(1)**,** 14176. doi: 10.1038/s41598-020-71142-3.

Verzhanskaya, T.Y., and Tarutta, E.P. (2017). Stabilizing effectiveness of orthokeratology and long-term minute-concentration atropine therapy in myopia (draft report). *Vestn. Oftalmol.* 133(5)**,** 43-48. doi: 10.17116/oftalma2017133543-48.

Vincent, S.J., Tan, Q., Ng, A.L.K., Cheng, G.P.M., Woo, V.C.P., and Cho, P. (2020). Higher order aberrations and axial elongation in combined 0.01% atropine with orthokeratology for myopia control. *Ophthalmic Physiol. Opt.* 40(6)**,** 728-737. doi: 10.1111/opo.12730.

Wan, L., Wei, C.C., Chen, C.S., Chang, C.Y., Lin, C.J., Chen, J.J., et al. (2018). The Synergistic Effects of Orthokeratology and Atropine in Slowing the Progression of Myopia. *J Clin Med* 7(9). doi: 10.3390/jcm7090259.

Wang, H.J. (2017). Efficacy of atropine and anisodamine eye drops for adolescent pseudomyopia. *International Eye Science* 17(3)**,** 519-521. doi: 10.3980/j.issn.1672-5123.2017.3.33.

Wang, M., Cui, C., Yu, S.A., Liang, L.L., Ma, J.X., and Fu, A.C. (2023). Effect of 0.02% and 0.01% atropine on ocular biometrics: A two-year clinical trial. *Front Pediatr* 11**,** 1095495. doi: 10.3389/fped.2023.1095495.

Wang, Y., Zhu, X., Xuan, Y., Wang, M., Zhou, X., and Qu, X. (2022). Short-Term Effects of Atropine 0.01% on the Structure and Vasculature of the Choroid and Retina in Myopic Chinese Children. *Ophthalmol. Ther.* 11(2)**,** 833-856. doi: 10.1007/s40123-022-00476-0.

Wang, Y.R., Bian, H.L., and Wang, Q. (2017). Atropine 0.5% eyedrops for the treatment of children with low myopia: A randomized controlled trial. *Medicine (Baltimore)* 96(27)**,** e7371. doi: 10.1097/md.0000000000007371.

Wang, Z., Hu, Y., and Zhen, M. (2021). Efficay and nursing observation of 0.01% atropine eye drops on the development of myopia in school-age children. *Journal of North Pharmacy* 18(01)**,** 67-68. doi: 10.3969/j.issn.1672-8351.2021.01.028.

Wu, P.C., Yang, Y.H., and Fang, P.C. (2011). The long-term results of using low-concentration atropine eye drops for controlling myopia progression in schoolchildren. *J. Ocul. Pharmacol. Ther.* 27(5)**,** 461-466. doi: 10.1089/jop.2011.0027.

Wu, T.E., Yang, C.C., and Chen, H.S. (2012). Does atropine use increase intraocular pressure in myopic children? *Optom. Vis. Sci.* 89(2)**,** E161-167.

Ye, L., Li, S., Shi, Y., Yin, Y., He, J., Zhu, J., et al. (2021). Comparisons of atropine versus cyclopentolate cycloplegia in myopic children. *Clin. Exp. Optom.* 104(2)**,** 143-150. doi: 10.1111/cxo.13128.

Ye, L., Shi, Y., Yin, Y., Li, S., He, J., Zhu, J., et al. (2020). Effects of Atropine Treatment on Choroidal Thickness in Myopic Children. *Invest. Ophthalmol. Vis. Sci.* 61(14)**,** 15. doi: 10.1167/iovs.61.14.15.

Zhang, X., Wang, Y., Zhou, X., and Qu, X. (2020). Analysis of Factors That May Affect the Effect of Atropine 0.01% on Myopia Control. *Front. Pharmacol.* 11. doi: 10.3389/fphar.2020.01081.

Zhao, H., Liu, Y., and Bai, F. (2015). Comparative Study on the 1-year Efficacy of 1% Atropine Sulfate Eye Gel and Orthokeratology in Preventing Myopia. *Medical Recapitulate* 21(01)**,** 166-169. doi: 10.3969/j.issn.1006-2084.2015.01.069.

Zhao, Q., and Hao, Q. (2021). Comparison of the Clinical Efficacies of 0.01% Atropine and Orthokeratology in Controlling the Progression of Myopia in Children. *Ophthalmic Epidemiol.* 28(5)**,** 376-382. doi: 10.1080/09286586.2021.1875010.

Zhao, W., Li, Z., Hu, Y., Jiang, J., Long, W., Cui, D., et al. (2021). Short-term effects of atropine combined with orthokeratology (ACO) on choroidal thickness. *Cont Lens Anterior Eye* 44(3)**,** 101348. doi: 10.1016/j.clae.2020.06.006.

Zheng, L., Tan, D., Zhang, M., Yang, Y., and Jin, W. (2022). Study on the eye safety of 0.01% Atropine Eye Drops for adolescent myopia control. *China Modern Medicine* 29(23)**,** 106-110. doi: 10.3969/j.issn.1674-4721.2022.23.028.

Zhou, H., Zhao, G., and Li, Y. (2022). Adjunctive effects of orthokeratology and atropine 0.01% eye drops on slowing the progression of myopia. *Clin. Exp. Optom.* 105(5)**,** 520-526. doi: 10.1080/08164622.2021.1943318.

**Supplementary Table 4.** **Baseline characteristics of the included studies.**

| Study | Study design | Country | Age (y) | Follow-up Duration (months) | Phase /group | Arm | Sample Size | Baseline Refraction (D) | Baseline Axial Length (mm) |
| --- | --- | --- | --- | --- | --- | --- | --- | --- | --- |
| Yen 1989(Yen et al., 1989) | RCT | Taiwan | 6–14 | 12 |  | 1% | 32 | –1.52 (0.96) | NA |
|  |  |  |  |  |  | Saline | 32 | –1.59 (0.92) | NA |
| Shih 1999(Shih et al., 1999) | RCT | Taiwan | 6–13 | 21 |  | 0.50% | 41 | –4.89 (2.06) | NA |
|  |  |  |  | 20 |  | 0.25% | 47 | –4.24 (1.74) | NA |
|  |  |  |  | 20 |  | 0.10% | 49 | –4.41 (1.47) | NA |
| Chua 2006 (ATOM)(Chua et al., 2006) | RCT | Singapore | 6–12 | 24 |  | 1% atropine sulfate | 166 | –3.36 (1.38) | 24.80 (0.83) |
|  |  |  |  |  |  | Vehicle (0.5% hydroxypropyl methylcellulose+1:10000 benzalkonium chloride) | 190 | –3.58 (1.17) | 24.80 (0.84) |
| Chia 2009 (ATOM)(Chia et al., 2009) | RCT | Singapore | 6–12 | 36 |  | 1% atropine sulfate | 166 | –3.36 (1.38) | 24.80 (0.83) |
|  |  |  |  |  |  | Vehicle (0.5% hydroxypropyl methylcellulose+1:10000 benzalkonium chloride) | 190 | –3.58 (1.17) | 24.80 (0.84) |
| Tong 2009 (ATOM)(Tong et al., 2009) | RCT | Singapore | 6–12 | 36 | cessation | 1% atropine sulfate | 158 | –3.36 (1.38) | 24.80 (0.83) |
|  |  |  |  |  |  | Vehicle (0.5% hydroxypropyl methylcellulose+1:10000 benzalkonium chloride) | 175 | –3.58 (1.17) | 24.80 (0.84) |
| Chia 2012 (ATOM2)(Chia et al., 2012) | RCT | Singapore | 6–12 | 24 |  | 0.50% | 139 | –4.70 (1.80) | 25.20 (0.90) |
|  |  |  |  |  |  | 0.10% | 141 | –4.80 (1.50) | 25.20 (0.80) |
|  |  |  |  |  |  | 0.01% | 75 | –4.50 (1.50) | 25.10 (1.00) |
| Chia 2014 (ATOM2)(Chia et al., 2014) | RCT | Singapore | 6–12 | 24-36 | cessation | 0.50% | 139 | –4.33 (1.83) | 25.14 (0.92) |
|  |  |  |  |  |  | 0.10% | 141 | –4.49 (1.45) | 25.13 (0.83) |
|  |  |  |  |  |  | 0.01% | 75 | –4.47 (1.50) | 25.17 (0.98) |
| Kumaran 2015 (ATOM)(Kumaran et al., 2015) | RCT | Singapore | 6–12 | 36 | cessation | 1% atropine sulfate | 147 | –3.36 (1.38) | 24.80 (0.83) |
|  |  |  |  |  |  | Vehicle (0.5% hydroxypropyl methylcellulose+1:10000 benzalkonium chloride) | 166 | –3.58 (1.17) | 24.80 (0.84) |
| Yi 2015(Yi et al., 2015) | RCT | China | 7–12 | 12 |  | 1% | 68 | –1.23 (0.32) | 23.75 (0.12) |
|  |  |  |  |  |  | Vehicle | 64 | –1.15 (0.30) | 23.72 (0.12) |
| Zhang 2019(Zhang et al., 2019) | RCT | China | 7–15 | 12 |  | 0.10% | 30 | –2.44 (1.13) | 24.71 (1.31) |
|  |  |  |  |  |  | 0.05% | 30 | –2.38 (1.10) | 24.46 (1.11) |
|  |  |  |  |  |  | 0.01% | 30 | –2.38 (1.19) | 24.69 (1.91) |
|  |  |  |  |  |  | No intervention | 30 | –2.09 (1.09) | 23.98 (1.15) |
| Yam 2019 (LAMP)(Yam et al., 2019) | RCT | Hong Kong, China | 4–12 | 12 | phase 1 | 0.05% | 102 | –3.98 (1.69) | 24.85 (0.90) |
|  |  |  |  |  |  | 0.025% | 91 | –3.71 (1.85) | 24.86 (0.95) |
|  |  |  |  |  |  | 0.01% | 97 | –3.77 (1.85) | 24.70 (0.99) |
|  |  |  |  |  |  | Placebo | 93 | –3.85 (1.95) | 24.82 (0.97) |
| Li 2020 (LAMP)(Li et al., 2020) | RCT | Hong Kong, China | 4–12 | 12 | phase 1 | 0.05% | 102 | –3.95 (1.64) | 24.86 (0.90) |
|  |  |  |  |  |  | 0.025% | 91 | –3.83 (1.81) | 24.92 (0.89) |
|  |  |  |  |  |  | 0.01% | 97 | –3.95 (1.90) | 24.79 (1.02) |
|  |  |  |  |  |  | Placebo | 93 | –4.10 (1.91) | 24.90 (0.99) |
| Yam 2020 (LAMP)(Yam et al., 2020) | RCT | Hong Kong, China | 4–12 | 12 | phase 2 | 0.05% | 93 | –3.93 (1.63) | 24.88 (0.91) |
|  |  |  |  |  |  | 0.025% | 86 | –3.88 (1.83) | 24.94 (0.90) |
|  |  |  |  |  |  | 0.01% | 91 | –3.99 (1.94) | 24.78 (1.02) |
| Wei 2020(Wei et al., 2020) | RCT | Beijing, China | 6–12 | 12 |  | 0.01% | 76 | –2.52 (1.33) | 24.50 (0.76) |
|  |  |  |  |  |  | Placebo | 83 | –2.64 (1.46) | 24.69 (0.97) |
| Zhu 2020(Zhu et al., 2020) | RCT | China | 6–12 | 24 |  | 1% | 262 | –3.82 (0.44) | 24.93 (0.21) |
|  |  |  |  |  |  | Placebo | 308 | –3.74 (0.51) | 24.91 (0.18) |
| Alam 2020(Alam et al., 2020) | RCT | Bangladesh | 6–18 | 12 |  | 0.01% | 24 | –3.00 (1.60) | 24.30 (1.00) |
|  |  |  |  |  |  | Artificial tear drops | 12 | –3.50 (1.60) | 24.60 (1.10) |
|  |  |  |  |  |  | Single-vision spectacles | 89 | –2.66 (1.39) | 24.54 (0.69) |
| Hieda 2021(Hieda et al., 2021) | RCT | Japan | 6–12 | 24 |  | 0.01% | 77 | –2.91 (1.30) | 24.43 (0.74) |
|  |  |  |  |  |  | Placebo | 81 | –2.98 (1.59) | 24.51 (0.78) |
| Zhao 2021(Zhao and Hao, 2021) | RCT | China | 5–14 | 12 |  | Spectacles+0.01% atropine | 20 | –1.98 (0.45) | 24.17 (0.68) |
|  |  |  |  |  |  | Spectacles | 20 | –1.93 (0.74) | 24.28 (0.83) |
| Moriche-Carretero 2021(Moriche-Carretero et al., 2021) | RCT | Span | 5–11 | 24 |  | 0.01% | 171 | –2.13 (0.63) | 24.22 (0.66) |
|  |  |  |  |  |  | No intervention | 168 | –2.16 (0.62) | 24.26 (0.91) |
| Yam 2022 (LAMP)(Yam et al., 2022) | RCT | Hong Kong, China | 4–12 | 36 | Phase 3 continued group | 0.05% | 45 | –3.93 (1.63) | 24.88 (0.91) |
|  |  |  |  |  |  | 0.025% | 39 | –3.88 (1.83) | 24.94 (0.90) |
|  |  |  |  |  |  | 0.01% | 43 | –3.99 (1.94) | 24.78 (1.02) |
|  |  |  |  |  | Phase 3 Washout group | 0.05% | 45 | –3.93 (1.63) | 24.88 (0.91) |
|  |  |  |  |  |  | 0.025% | 39 | –3.88 (1.83) | 24.94 (0.90) |
|  |  |  |  |  |  | 0.01% | 43 | –3.99 (1.94) | 24.78 (1.02) |
| Chan 2022(Chan et al., 2022) | RCT | China | 7–10 | 18 |  | 0.01% | 34 | –1.88 (1.08) | 24.17 (0.79) |
|  |  |  |  |  |  | Placebo | 27 | –1.74 (0.71) | 24.09 (0.74) |
| Bukhari 2022(Bukhari et al., 2022) | RCT | China | 6-12 | 12 |  | 0.01% | 76 | –2.55 (1.29) | 24.58 (0.80) |
|  |  |  |  |  |  | Placebo | 83 | –2.68 (1.43) | 24.64 (0.98) |
| Lee 2022(Lee et al., 2022) | RCT | China | 6-16 | 24 |  | 0.01% | 94 | –3.56 (0.51) | 24.70 (0.15) |
|  |  |  |  |  |  | Placebo | 37 | –3.92 (1.01) | 24.54 (0.64) |
| Sen 2022(Sen et al., 2022) | RCT | Australia | 5-15 | 24 |  | 0.01% | 72 | –3.92 (1.01) | 24.54 (0.64) |
|  |  |  |  |  |  | Placebo | 73 | –4.05 (1.25) | 24.58 (0.79) |
| Sharma 2022(Sharma et al., 2022) | RCT | India | 5-12 | 12 |  | 0.01% | 50 | –3.43 (3.32) | 24.36 (1.68) |
|  |  |  |  |  |  | Placebo | 50 | –2.91 (2.74) | 24.11 (1.32) |
| Xu 2022(Xu et al., 2022) | RCT | India | 8-12 | 24 |  | 0.01%+spectacles | 31 | NA | NA |
|  |  |  |  |  |  | Placebo+spectacles | 30 | NA | NA |
| Liang 2023-1(Liang et al., 2023) | RCT | China | 6-12 | 12 |  | 0.01% | 76 | –2.69 (1.27) | 24.64 (0.78) |
|  |  |  |  |  |  | Placebo | 83 | –2.85 (1.45) | 24.73 (0.97) |
| Liang 2023-2(Liang et al., 2023) | RCT | China | 6-12 | 12 |  | 0.01% | 76 | –2.29 (1.22) | 24.47 (0.80) |
|  |  |  |  |  |  | Placebo | 83 | –2.41 (1.53) | 24.54 (0.99) |
| Wang 2022(Wang et al., 2022) | cohort | China | 6–12 | 24 | Phase 2 | 0.02%+single vision | 105 | –2.81 (1.47) | NA |
|  |  |  |  |  |  | 0.01%+single vision | 106 | –2.76 (1.56) | NA |
|  |  |  |  |  |  | Single vision spectacles | 89 | –2.66 (1.39) | NA |
| Chaurasia 2022(Chaurasia et al., 2022) | cohort | India | 6–16 | 12 |  | 0.01% on right eyes | 40 | –3.04 (1.36) | 24.56 (0.33) |
|  |  |  |  |  |  | 0.5% carboxymethyl cellulose drops in left eyes | 40 | –3.07 (1.32) | 24.52 (0.31) |
| Jiang 2018(Jiang, 2018) | cohort | China | 8–16 | 18 |  | 0.01% atropine | 40 | –2.85 (0.54) | 23.93 (1.07) |
|  |  |  |  |  |  | spectacles | 40 | –2.98 (0.86) | 23.97 (1.22) |
| Lee 2016(Lee et al., 2016) | cohort | China | 6–12 | 12 |  | 0.25% | 12 | –1.45 (0.69) | NA |
|  |  |  |  |  |  | 0.125% | 32 | –1.22 (0.55) | NA |
|  |  |  |  |  |  | spectacles | 12 | –1.45 (1.00) | NA |
| Liu 2021(Liu et al., 2021) | cohort | China | 8–14 | 12 |  | 0.01% atropine | 40 | –2.02 (1.50) | NA |
|  |  |  |  |  |  | spectacles | 40 | –2.18 (1.07) | NA |
|  |  |  |  |  |  | spectacles | 50 | –2.96 (0.34) | 24.31 (1.24) |
| Zhao 2022(Zhao et al., 2022) | cohort | China | 6–14 | 12 |  | 0.005% atropine+spectacles | 50 | –1.49 (0.59) | 24.06 (0.54) |
|  |  |  |  |  |  | spectacles | 49 | –1.39 (0.47) | 24.13 (0.59) |
| Zheng 2020(Zheng et al., 2020) | cohort | China | 7–13 | 12 |  | 0.01% | 23 | –4.12 (1.57) | 25.06 (1.17) |
|  |  |  |  |  |  | spectacles | 25 | –3.72 (1.57) | 25.07 (0.93) |
| Zhu 2021(Zhu et al., 2021) | cohort | China | 8–12 | 24 |  | 0.05% | 93 | –3.29 (0.22) | 23.75 (0.22) |
|  |  |  |  |  |  | 0.3% Sodium hyaluronate eye drops | 95 | –3.32 (0.52) | 23.68 (0.19) |
| Liu 2020(Liu et al., 2020) | cohort | China | 7–17 | 12 |  | 0.01% | 31 | –2.00 (0.80) | 24.00 (0.70) |
|  |  |  |  |  |  | spectacles | 30 | –1.90 (0.80) | 23.90 (0.80) |
| Cai 2021(Cai et al., 2021) | cohort | China | 6–12 | 24 |  | 1.00% | 66 | –3.05 (1.55) | NA |
|  |  |  |  |  |  | 0.025% | 68 | –3.48 (1.43) | NA |
|  |  |  |  |  |  | 0.01% | 79 | –3.52 (1.37) | NA |
|  |  |  |  |  |  | no intervention | 76 | –3.11 (1.48) | NA |
| Agarwal 2022(Agarwal et al., 2022) | cohort | India | 6-16 | 24 |  | 0.01% | 37 | –10.60 (2.50) | NA |
|  |  |  |  |  |  | Single vision spectacles | 23 | –11.40 (3.60) | NA |
| Nucci 2023(Nucci et al., 2023) | cohort | Italy | 6-18 | 12 |  | 0.01% | 53 | –1.56 (0.69) | 24.64 (0.89) |
|  |  |  |  |  |  | Single vision spectacles | 32 | –1.54 (0.74) | 24.64 (0.79) |
| Fu 2020(Fu et al., 2020) | cohort | China | 6–14 | 12 | Phase 1 | 0.02% | 117 | –2.76 (1.47) | 24.60 (0.72) |
|  |  |  |  |  |  | 0.01% | 119 | –2.70 (1.64) | 24.58 (0.74) |
|  |  |  |  |  |  | Single-vision spectacles | 100 | –2.68 (1.42) | 24.55 (0.71) |
| Cui 2021(Cui et al., 2021) | cohort | China | 6–14 | 24 | Phase 2 | 0.02%+best-corrected spectacles | 105 | –2.81 (1.47) | 24.61 (0.69) |
|  |  |  |  |  |  | 0.01%+best-corrected spectacles | 106 | –2.76 (1.56) | 24.60 (0.72) |
| Diaz–Llopis 2018(Diaz-Llopis and Pinazo-Durán, 2018) | cohort | Spain | 9–12 | 60 |  | 0.01% | 100 | –1.10 (0.50) | NA |
|  |  |  |  |  |  | Control | 100 | –1.20 (0.40) | NA |
| Fan 2007(Fan et al., 2007) | cohort | China | 5–10 | 12 |  | 1%+Photochromic progressive glasses glasses | 23 | –5.18 (2.05) | 25.06(1.03) |
|  |  |  |  |  |  | no intervention | 23 | –5.12 (2.33) | 24.85(0.78) |
| Sacchi 2019(Sacchi et al., 2019) | cohort | Italy | 5–16 | At least 12 |  | 0.01% | 52 | –3.00 (2.23) | NA |
|  |  |  |  |  |  | control | 50 | –2.63 (2.68) | NA |

**Reference:**

Agarwal, P., Khurana, A., Maan, V., Sutar, S., and Chauhan, L. (2022). Role of 0.01% atropine in high myopic children of Moradabad, India (RAMCOM Study). *Indian J. Ophthalmol.* 70(12)**,** 4400-4404. doi: 10.4103/ijo.IJO_679_22.

Alam, A.R.M., Hossain, M.S., and Islam, M.S. (2020). Topical atropine in retarding myopia progression and axial length growth in children with myopia. *Bangabandhu Sheikh Mujib Med Univ J* 13**,** 111-114.

Bukhari, J., Wei, S.F., Li, S.M., An, W.Z., Du, J.L., Liang, X.T., et al. (2022). Effect of 0.01% atropine eyedrops on intraocular pressure in schoolchildren: a randomized clinical trial. *Int J Ophthalmol* 15(9)**,** 1431-1436. doi: 10.18240/ijo.2022.09.04.

Cai, J., Li, J., Xiong, Y., Gong, Y., and Sun, Z. (2021). Effects of different concentrations of atropine in the control of myopia in children. *Journal of Nantong University (Medical Sciences)* 41(02)**,** 191-193. doi: 10.16424/j.cnki.cn32-1807/r.2021.02.024.

Chan, H.H.L., Choi, K.Y., Ng, A.L.K., Choy, B.N.K., Chan, J.C.H., Chan, S.S.H., et al. (2022). Efficacy of 0.01% atropine for myopia control in a randomized, placebo-controlled trial depends on baseline electroretinal response. *Sci. Rep.* 12(1)**,** 11588. doi: 10.1038/s41598-022-15686-6.

Chaurasia, S., Negi, S., Kumar, A., Raj, S., Kaushik, S., Optom, R.K.M., et al. (2022). Efficacy of 0.01% low dose atropine and its correlation with various factors in myopia control in the Indian population. *Sci. Rep.* 12(1)**,** 7113. doi: 10.1038/s41598-022-10079-1.

Chia, A., Chua, W.H., Cheung, Y.B., Wong, W.L., Lingham, A., Fong, A., et al. (2012). Atropine for the treatment of childhood myopia: safety and efficacy of 0.5%, 0.1%, and 0.01% doses (Atropine for the Treatment of Myopia 2). *Ophthalmology* 119(2)**,** 347-354. doi: 10.1016/j.ophtha.2011.07.031.

Chia, A., Chua, W.H., and Tan, D. (2009). Effect of topical atropine on astigmatism. *Br. J. Ophthalmol.* 93(6)**,** 799-802. doi: 10.1136/bjo.2008.147421.

Chia, A., Chua, W.H., Wen, L., Fong, A., Goon, Y.Y., and Tan, D. (2014). Atropine for the treatment of childhood myopia: changes after stopping atropine 0.01%, 0.1% and 0.5%. *Am. J. Ophthalmol.* 157(2)**,** 451-457.e451. doi: 10.1016/j.ajo.2013.09.020.

Chua, W.H., Balakrishnan, V., Chan, Y.H., Tong, L., Ling, Y., Quah, B.L., et al. (2006). Atropine for the treatment of childhood myopia. *Ophthalmology* 113(12)**,** 2285-2291. doi: 10.1016/j.ophtha.2006.05.062.

Cui, C., Li, X., Lyu, Y., Wei, L., Zhao, B., Yu, S., et al. (2021). Safety and efficacy of 0.02% and 0.01% atropine on controlling myopia progression: a 2-year clinical trial. *Sci. Rep.* 11(1)**,** 22267. doi: 10.1038/s41598-021-01708-2.

Diaz-Llopis, M., and Pinazo-Durán, M.D. (2018). Superdiluted atropine at 0.01% reduces progression in children and adolescents. A 5 year study of safety and effectiveness. *Arch Soc Esp Oftalmol (Engl Ed)* 93(4)**,** 182-185. doi: 10.1016/j.oftal.2017.12.015.

Fan, D.S., Lam, D.S., Chan, C.K., Fan, A.H., Cheung, E.Y., and Rao, S.K. (2007). Topical atropine in retarding myopic progression and axial length growth in children with moderate to severe myopia: a pilot study. *Jpn. J. Ophthalmol.* 51(1)**,** 27-33. doi: 10.1007/s10384-006-0380-7.

Fu, A., Stapleton, F., Wei, L., Wang, W., Zhao, B., Watt, K., et al. (2020). Effect of low-dose atropine on myopia progression, pupil diameter and accommodative amplitude: low-dose atropine and myopia progression. *Br. J. Ophthalmol.* 104(11)**,** 1535-1541. doi: 10.1136/bjophthalmol-2019-315440.

Hieda, O., Hiraoka, T., Fujikado, T., Ishiko, S., Hasebe, S., Torii, H., et al. (2021). Efficacy and safety of 0.01% atropine for prevention of childhood myopia in a 2-year randomized placebo-controlled study. *Jpn. J. Ophthalmol.* 65(3)**,** 315-325. doi: 10.1007/s10384-021-00822-y.

Jiang, J. (2018). Effect of orthokeratology, low concentration atropine and frame glasses on juvenile myopia prevention and control. *International Eye Science* 18(7)**,** 1349-1352. doi: 10.3980/j.issn.1672-5123.2018.7.46.

Kumaran, A., Htoon, H.M., Tan, D., and Chia, A. (2015). Analysis of Changes in Refraction and Biometry of Atropine- and Placebo-Treated Eyes. *Invest. Ophthalmol. Vis. Sci.* 56(9)**,** 5650-5655. doi: 10.1167/iovs.14-14716.

Lee, C.Y., Sun, C.C., Lin, Y.F., and Lin, K.K. (2016). Effects of topical atropine on intraocular pressure and myopia progression: a prospective comparative study. *BMC Ophthalmol.* 16**,** 114. doi: 10.1186/s12886-016-0297-y.

Lee, S.S., Lingham, G., Blaszkowska, M., Sanfilippo, P.G., Koay, A., Franchina, M., et al. (2022). Low-concentration atropine eyedrops for myopia control in a multi-racial cohort of Australian children: A randomised clinical trial. *Clin Exp Ophthalmol* 50(9)**,** 1001-1012. doi: 10.1111/ceo.14148.

Li, F.F., Kam, K.W., Zhang, Y., Tang, S.M., Young, A.L., Chen, L.J., et al. (2020). Differential Effects on Ocular Biometrics by 0.05%, 0.025%, and 0.01% Atropine: Low-Concentration Atropine for Myopia Progression Study. *Ophthalmology* 127(12)**,** 1603-1611. doi: 10.1016/j.ophtha.2020.06.004.

Liang, X., Wei, S., Li, S.M., An, W., Du, J., Sun, Y., et al. (2023). Effect of Atropine 0.01% Eye Drops on the Difference in Refraction and Axial Length Between Right and Left Eye. *Ophthalmic Res.* doi: 10.1159/000528878.

Liu, S., Wang, B., Wang, G.J., and Dong, J. (2021). Effects of different intervention methods on regulatory parameters and diopter of myopic children. *International Eye Science* 21(11)**,** 1870-1874. doi: 10.3980/j.issn.1672-5123.2021.11.07.

Liu, Z., Han, Y., Gao, N., and Liu, Y. (2020). Analysis of the efficacy of 0.01% atropine in the control of juvenile myopia. *Shanxin Med J* 49(05)**,** 591-592. doi: 10.3969/j.issn.0253-9926.2020.05.039.

Moriche-Carretero, M., Revilla-Amores, R., Diaz-Valle, D., Morales-Fernández, L., and Gomez-de-Liaño, R. (2021). Myopia progression and axial elongation in Spanish children: Efficacy of atropine 0.01% eye-drops. *J. Fr. Ophtalmol.* 44(10)**,** 1499-1504. doi: 10.1016/j.jfo.2021.07.005.

Nucci, P., Lembo, A., Schiavetti, I., Shah, R., Edgar, D.F., and Evans, B.J.W. (2023). A comparison of myopia control in European children and adolescents with defocus incorporated multiple segments (DIMS) spectacles, atropine, and combined DIMS/atropine. *PLoS One* 18(2)**,** e0281816. doi: 10.1371/journal.pone.0281816.

Sacchi, M., Serafino, M., Villani, E., Tagliabue, E., Luccarelli, S., Bonsignore, F., et al. (2019). Efficacy of atropine 0.01% for the treatment of childhood myopia in European patients. *Acta Ophthalmol. (Copenh.)* 97(8)**,** e1136-e1140. doi: 10.1111/aos.14166.

Sen, S., Yadav, H., Jain, A., Verma, S., and Gupta, P. (2022). Effect of atropine 0.01% on progression of myopia. *Indian J. Ophthalmol.* 70(9)**,** 3373-3376. doi: 10.4103/ijo.IJO_256_22.

Sharma, I., Das, G.K., Rohatgi, J., Sahu, P.K., Chhabra, P., and Bhatia, R. (2022). Low Dose Atropine in Preventing the Progression of Childhood Myopia: A Randomised Controlled Trial. *Curr. Eye Res.***,** 1-6. doi: 10.1080/02713683.2022.2162925.

Shih, Y.F., Chen, C.H., Chou, A.C., Ho, T.C., Lin, L.L., and Hung, P.T. (1999). Effects of different concentrations of atropine on controlling myopia in myopic children. *J. Ocul. Pharmacol. Ther.* 15(1)**,** 85-90. doi: 10.1089/jop.1999.15.85.

Tong, L., Huang, X.L., Koh, A.L., Zhang, X., Tan, D.T., and Chua, W.H. (2009). Atropine for the treatment of childhood myopia: effect on myopia progression after cessation of atropine. *Ophthalmology* 116(3)**,** 572-579. doi: 10.1016/j.ophtha.2008.10.020.

Wang, M., Cui, C., Sui, Y., Yu, S.A., Ma, J.X., and Fu, A.C. (2022). Effect of 0.02% and 0.01% atropine on astigmatism: a two-year clinical trial. *BMC Ophthalmol.* 22(1)**,** 161. doi: 10.1186/s12886-022-02385-z.

Wei, S., Li, S.M., An, W., Du, J., Liang, X., Sun, Y., et al. (2020). Safety and Efficacy of Low-Dose Atropine Eyedrops for the Treatment of Myopia Progression in Chinese Children: A Randomized Clinical Trial. *JAMA Ophthalmol* 138(11)**,** 1178-1184. doi: 10.1001/jamaophthalmol.2020.3820.

Xu, S., Li, Z., Zhao, W., Zheng, B., Jiang, J., Ye, G., et al. (2022). Effect of atropine, orthokeratology and combined treatments for myopia control: a 2-year stratified randomised clinical trial. *Br. J. Ophthalmol.* doi: 10.1136/bjo-2022-321272.

Yam, J.C., Jiang, Y., Tang, S.M., Law, A.K.P., Chan, J.J., Wong, E., et al. (2019). Low-Concentration Atropine for Myopia Progression (LAMP) Study: A Randomized, Double-Blinded, Placebo-Controlled Trial of 0.05%, 0.025%, and 0.01% Atropine Eye Drops in Myopia Control. *Ophthalmology* 126(1)**,** 113-124. doi: 10.1016/j.ophtha.2018.05.029.

Yam, J.C., Li, F.F., Zhang, X., Tang, S.M., Yip, B.H.K., Kam, K.W., et al. (2020). Two-Year Clinical Trial of the Low-Concentration Atropine for Myopia Progression (LAMP) Study: Phase 2 Report. *Ophthalmology* 127(7)**,** 910-919. doi: 10.1016/j.ophtha.2019.12.011.

Yam, J.C., Zhang, X.J., Zhang, Y., Wang, Y.M., Tang, S.M., Li, F.F., et al. (2022). Three-Year Clinical Trial of Low-Concentration Atropine for Myopia Progression (LAMP) Study: Continued Versus Washout: Phase 3 Report. *Ophthalmology* 129(3)**,** 308-321. doi: 10.1016/j.ophtha.2021.10.002.

Yen, M.Y., Liu, J.H., Kao, S.C., and Shiao, C.H. (1989). Comparison of the effect of atropine and cyclopentolate on myopia. *Ann. Ophthalmol.* 21(5)**,** 180-182, 187.

Yi, S., Huang, Y., Yu, S.Z., Chen, X.J., Yi, H., and Zeng, X.L. (2015). Therapeutic effect of atropine 1% in children with low myopia. *J. AAPOS* 19(5)**,** 426-429. doi: 10.1016/j.jaapos.2015.04.006.

Zhang, L., Liu, H., Zhu, G., Xiang, G., Wen, X., and Huang, H. (2019). Effect of prevention and control and safety of different low concentrations of atropine preparations for myopia in adolescents. *IMHGN* 25(24)**,** 4002-4006. doi: 10.3760/cma.j.issn.1007-1245.2019.24.009.

Zhao, B.X., Zhang, A.F., Cui, C., Wei, L., Li, B.B., Pang, X.N., et al. (2022). Safety and efficacy of 0.005% Atropine eye drops on myopia progression in children with low myopia. *International Eye Science* 22(3)**,** 388-393. doi: 10.3980/j.issn.1672-5123.2022.3.07.

Zhao, Q., and Hao, Q. (2021). Clinical efficacy of 0.01% atropine in retarding the progression of myopia in children. *Int. Ophthalmol.* 41(3)**,** 1011-1017. doi: 10.1007/s10792-020-01658-0.

Zheng, J., Liao, R., Feng, L., and He, X. (2020). Effect of 0.01% Atropine eye drops on controlling myopia and accommodation functionin adolescents. *J Clin Ophthalmol* 28(6)**,** 533-537. doi: 10.3969/j.issn.1006-8422.2020.06.013.

Zhu, Q., Tang, Y., Guo, L., Tighe, S., Zhou, Y., Zhang, X., et al. (2020). Efficacy and Safety of 1% Atropine on Retardation of Moderate Myopia Progression in Chinese School Children. *Int. J. Med. Sci.* 17(2)**,** 176-181. doi: 10.7150/ijms.39365.

Zhu, Q., Zhou, Y., Zhang, X., Zhang, J., and Hu, M. (2021). Efficacy and safety of 0.05% atropine eye drops for retarding myopia progression in Chinese primary school students. *Chin J Sch Health* 42(2)**,** 170-176. doi: 10.16835/j.cnki.1000-9817.2021.02.003.

**Supplementary Table 5.** **Quality assessment of RCTs included in the meta-analysis according to the Cochrane Collaboration Tool.**

1. **Risk of bias graph:**
2. **Risk of bias summary:**

**Supplementary Table 6.** **Quality assessment of cohort studies included and excluded in the meta-analysis using Newcastle-Ottawa Quality Assessment Scale.**

| first author | year | Representativeness of the exposed cohort | Selection of the non- exposed cohort | Ascertainment of exposure | Demonstration that outcome of interest was not present at start of study | Comparability of cohorts on the basis of the design or analysis | Assessment of outcome | Was follow-up long enough for outcomes to occur | Adequacy of follow up of cohorts | Score |
| --- | --- | --- | --- | --- | --- | --- | --- | --- | --- | --- |
| **Included studies** | |  |  |  |  |  |  |  |  |  |
| Chaurasia 2022 | 2022 | ***** | ***** | ***** | ***** | ****** | ***** | ***** | ***** | 9 |
| Jiang 2018 | 2018 | ***** | ***** | ***** | **-** | ****** | ***** | ***** | ***** | 8 |
| Lee 2016 | 2016 | ***** | ***** | ***** | ***** | ****** | ***** | ***** | ***** | 9 |
| Liu 2021 | 2021 | ***** | ***** | ***** | **-** | ****** | ***** | ***** | ***** | 8 |
| Zhao 2022 | 2022 | ***** | ***** | ***** | ***** | ****** | ***** | ***** | ***** | 9 |
| Zheng 2020 | 2020 | ***** | ***** | ***** | **-** | ****** | ***** | ***** | ***** | 9 |
| Zhu 2021 | 2021 | ***** | ***** | ***** | ***** | ****** | ***** | ***** | ***** | 9 |
| Liu 2020 | 2020 | ***** | ***** | ***** | ***** | ****** | ***** | ***** | ***** | 9 |
| Cai 2021 | 2021 | ***** | ***** | ***** | ***** | ****** | ***** | ***** | ***** | 9 |
| Agarwal 2022 | 2022 | ***** | ***** | ***** | **-** | ****** | ***** | ***** | ***** | 8 |
| Nucci 2023 | 2023 | **-** | ***** | ***** | ***** | ****** | ***** | ***** | ***** | 8 |
| Fu 2020 | 2020 | ***** | ***** | ***** | ***** | ****** | ***** | ***** | ***** | 9 |
| Cui 2021 | 2021 | ***** | ***** | ***** | ***** | ****** | ***** | ***** | ***** | 9 |
| Wang 2022 | 2022 | ***** | ***** | ***** | ***** | ****** | ***** | ***** | ***** | 9 |
| Diaz-LIopis 2018 | 2018 | ***** | ***** | ***** | ***** | ****** | ***** | ***** | **-** | 8 |
| Fan 2007 | 2007 | ***** | ***** | - | ***** | ****** | ***** | ***** | ***** | 8 |
| Sacchi 2019 | 2019 | ***** | ***** | - | ***** | ***** | ***** | ***** | ***** | 7 |
| **Excluded studies** | | | | | | | | | | |
| Erdinest 2022 | 2022 | **-** | ***** | * | **-** | ***** | ***** | ***** | ***** | 6 |
| ***** According to the Quality Assessment Scale items, stars of 0–3, 4–6, 7–9 were considered as low, moderate, and high quality, respectively. 1 “*” represents 1 score. | | | | | | | | | | |

**Supplementary Table 7.** **Meta-regression analysis of mean difference in refraction change and axial length change.**

| **Outcome** | **Variable** | **Pooled estimate (95% CI)** | **P** |
| --- | --- | --- | --- |
| **Refraction change** | Publication year**^‡^** | -0.22 (-2.64 to 0.20) | 0.29 |
|  | Baseline mean refractive error**^§^** | -0.02 (-0.23 to 0.19) | 0.84 |
|  | Sample size**^\|\|^** | -0.14 (-0.35 to 0.06) | 0.17 |
|  | Ethnicity**^¶^** | -0.04 (-0.35 to 0.26) | 0.77 |
|  | Risk of bias**^**^** | 0.15 (-0.35 to -0.65) | 0.55 |
| **Axial length change** | Baseline mean refractive error**^§^** | -0.01 (-0.12 to 0.11) | 0.90 |
|  | Sample size**^\|\|^** | -0.06 (-0.07 to 0.20) | 0.36 |
|  | Ethnicity**^¶^** | 0.10 (-0.16 to 0.36) | 0.45 |
|  | Risk of bias**^**^** | -0.05 (-0.39 to 0.49) | 0.81 |

*****P<0.05 indicates statistically significant. **†**CI= confidence interval. **‡**Publishing before 2000. **§**Baselinee mean refractive error less than -4 diopters. **||** Sample size fewer than 50 participants. **¶**Conducted in Asian patients. ******High risk of bias.
